# Income–Well‐Being Gradient in Sickness and Health

**DOI:** 10.1002/hec.70063

**Published:** 2025-11-19

**Authors:** Ohto Kanninen, Petri Böckerman, Ilpo Suoniemi

**Affiliations:** ^1^ Labour Institute for Economic Research LABORE and University of Helsinki Helsinki Finland; ^2^ University of Jyväskylä Labour Institute for Economic Research LABORE and IZA Institute of Labor Economics Helsinki Finland; ^3^ Labour Institute for Economic Research LABORE Helsinki Finland

**Keywords:** life satisfaction, optimal benefit, risk aversion, sickness absence, social insurance, state dependence

## Abstract

We propose a method for studying the value of insurance. For this purpose, we analyze the well‐being of the same individuals, comparing sick and healthy years, using German panel survey data on life satisfaction. We impose structure on the income–well‐being gradient by fitting a flexible utility function to the data, focusing on the differences in marginal utility in the sick and the healthy states. Notably, our empirical specification allows for a “fixed cost of sickness.” We find a higher marginal utility of income in the sick state. We use our estimates to gauge the value of sickness insurance for Baily‐Chetty–type optimal policy calculations.

## 1 Introduction

Social insurance that protects workers against unemployment, sickness, and old age accounts for a significant share of government expenditures. The potential negative moral hazard effects of these programs on workers' labor supply have been the topic of an ever‐growing body of literature (see, e.g., the reviews by Chetty and Finkelstein 2013; Schmieder and Von Wachter 2016). The optimal social insurance—characterized by the Baily‐Chetty formula (Baily 1978; Chetty 2006)—balances the moral hazard cost of providing insurance with its value to the insured. The value of insurance is captured by the marginal rate of substitution (MRS) between in‐work and out‐of‐work consumption. However, less evidence is available regarding the value of social insurance relative to its costs. The main reason for this is that social insurance programs, such as sickness insurance (SI), are mandated, so they leave no opportunities to study the benefits through choices. We focus on sickness absence, because it causes a loss of nearly 10% of annual working days in some OECD countries (DICE Database 2017).

The traditional approach in the literature, as implemented by Gruber (1997) in the context of unemployment (UI), is to focus on consumption smoothing. The estimated drop in consumption in response to an adverse event can be scaled by the workers' risk aversion to obtain an estimate of the value of providing additional insurance against that event, assuming a state‐independent utility. Kolsrud et al. (2018) introduced an alternative method for estimating the value of unemployment insurance (UI) using consumption data. They considered the difference in marginal propensity to consume (MPC) when unemployed versus employed, which allowed them to identify the difference in shadow prices to smooth consumption in the respective states.

We explore a third approach that extends the literature in two important ways. Our first contribution is to use panel data on subjective well‐being to estimate a “fixed cost of sickness”, which allows for the estimation of the marginal utility of income in different health states. Our second contribution is to inform optimal sickness insurance design by incorporating the estimated fixed cost of sickness directly into the Baily‐Chetty formula.

To estimate fixed cost of sickness, we impose structure on the income–well‐being gradient in the sick and healthy states by fitting a flexible, nonlinear, state‐dependent utility function. We compare our results to those of a simplified Finkelstein et al. (2013) type loglinear fixed effects model. The main benefit of our approach is that the cost of sickness is denoted in monetary terms and that it has more statistical power than the loglinear model. Our approach also has the advantage of not imposing strong assumptions on the functional form of the income–well‐being gradient. However, the benefit of the loglinear model is its ability to account for individual fixed effects.

In principle, one can infer risk preferences and state dependence empirically by studying individuals' revealed preferences (e.g., Cohen and Einav 2007) or by analyzing subjective well‐being in different states (Finkelstein et al. 2009; Mata et al. 2018). We take the latter approach.

In our empirical utility function fits, we allow for a horizontal shift parameter corresponding to a “fixed cost of sickness,” which affects the utility gain of consumption smoothing and could, in principle, be of either sign. If high, it can potentially be a major determinant of the shift in relative risk aversion and marginal utility in the sick state compared to the employed state. We also allow for level shifts in utility between states. A level shift does not affect marginal utility and thus does not affect policy. Our method leads to a domain‐focused view of risk.

**FIGURE 1:**
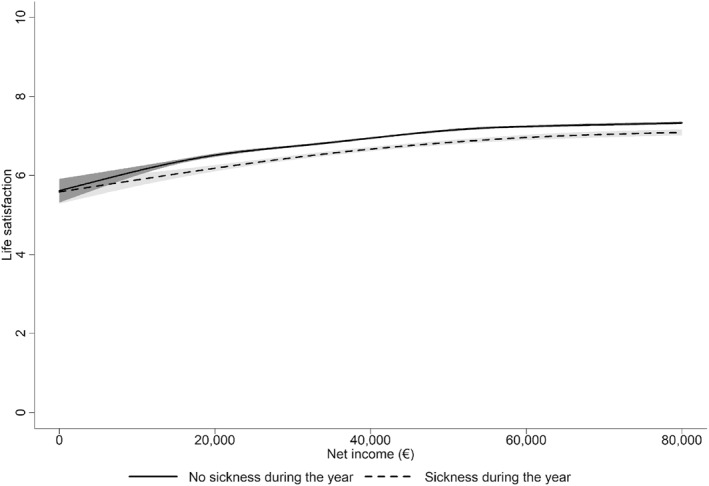
Spline fit of the income–well‐being gradient in Germany split by sickness states. The estimates are cubic splines with six knots estimated using the R package “bigsplines” for a cross‐section using the following parameter values; unrounded, no manual tuning, and unique knots assuming Gaussian standard errors. The gray area around the curves represents the 95% confidence interval. Sample size: 56,268 years‐person observations with no sickness during the year, 10,961 years‐person observations with sickness during the year.

A mapping of net income on life satisfaction or the income–well‐being gradient for individuals who alternate between sickness states, as shown in Figure 1, immediately reveals two qualitative and systematic patterns concerning the sickness absence state. First, life satisfaction in the sick state is lower, conditional on income. Second, assuming that the relationship is causal, the marginal utility in the sick state is higher (i.e., it shows a positive state dependence). Consequently, a convergence in life satisfaction is also evident between the two states at higher levels of income.

Our analysis assumes that life satisfaction approximates utility. The fitted parametric form utilizes minimal restrictive assumptions and allows us to identify the relevant parameters of the utility function, including the term related to the fixed cost of sickness. The estimates show that the fixed cost is a parsimonious way to model state dependence in marginal utility and to capture the salient features in Figure 1, except at very low incomes. We employ cross‐sectional and panel variation when estimating the utility function, and the estimates are biased by omitted variables, such as education or reverse causality from well‐being to income. However, the estimated cost of sickness is identified using within‐individual variation in the sickness state. For policy purposes, we are interested in how sickness is associated with shifts in the marginal utility compared to the marginal utility of the same individuals in full health. Even if sickness is caused, for example, by other shocks in life that also directly affect marginal utility, a sickness insurance policy should be optimized for the new marginal utility, independent of the mechanisms causing the change.

The empirical findings support our method of incorporating the standard utility theory and insights from recent behavioral economics research. The estimate of the utility function under the healthy state conforms to the standard utility function, again with possible endogeneity bias. However, allowing for and empirically finding a significant difference in the utility curve for those who are on sick leave emphasizes the importance of state dependence on risk. A limitation of our analysis is the fact that we can only identify sickness spells at the annual level and remain unaware of whether life satisfaction is reported during the sickness spell. This limitation leads to measurement error, and we study the importance of this error using alternative specifications.

Economic theory argues that the optimal policy rule in insurance schemes constitutes a trade‐off between benefits (i.e., the consumption smoothing effect) and costs (i.e., due to hidden actions; the moral hazard effect). The canonical Baily‐Chetty formula is based on a state‐independent utility function (Baily 1978; Chetty 2006, 2008). We extend the canonical model and show that the standard measure of relative risk aversion is empirically lacking and that the fixed cost of sickness explains a substantial part of the effect of sickness on marginal utility, that is, the utility function is state‐dependent. The last point also implies that social insurance schemes must be calibrated according to our best empirical understanding of utility and risk in each policy domain.

Our results are relevant to the core features of sickness absence policies. Three points stand out. First, we contribute to the literature on state dependence by showing that marginal utility is higher in the sick state, conditional on income. Traditionally, the focus has been on consumption smoothing because consumption and the curvature of the utility function are sufficient statistics in the received view to characterize the benefit of the insurance. However, a more subtle understanding of marginal utility of the sick versus employed states is required. Second, we establish that—contrary to conventional wisdom—relative risk aversion in the employed state increases with income. This stems from the negative estimated horizontal shift or “institutions” parameter. Third, for lower‐income individuals, the fixed cost of sickness is the main reason to insure against sickness, since their relative risk aversion is low due to the baseline protection given by the institutions. Low‐income individuals benefit mainly from being insured against the domain‐specific fixed cost of sickness.

Concerning sickness, the assumption of state independence of the utility function has been challenged by previous contributions. The empirical literature provides conflicting results concerning whether marginal utility is higher or lower for the sick population (see Finkelstein et al. 2009, 117; Viscusi and Evans 1990; Viscusi 2019). The empirical literature on insurance choice in economics, along with the psychological literature, has found that risk‐taking is highly domain specific (Einav et al. 2012; Weber et al. 2002), which is consistent with state‐dependent utility functions. We thoroughly compare our results to a simplified version of the loglinear specification of Finkelstein et al. (2013).

The paper is structured as follows. Section 2 discusses the key theoretical aspects of an optimal sickness insurance system. Section 3 describes the data and characterizes the utility function and empirical estimation methods. Section 4 reports the estimation results. The last section concludes the paper.

## 2 Optimal Sickness Insurance

The goal of social insurance is to transfer resources from states of the world with low marginal utility to states of the world with high marginal utility. We apply and develop the Baily‐Chetty approach to sickness insurance (Baily 1978; Chetty 2006; Chetty and Finkelstein 2013).^1^ The theoretical model describes a welfare‐maximizing social planner's optimal choice of sickness benefits and taxes, given the costs and benefits of a higher sickness allowance for a utility‐maximizing representative agent who chooses the length of the sickness absence spell. Following the literature, the agent cannot borrow. Chetty (2008) studies the implications of liquidity constraints on optimal unemployment insurance. The costs of higher replacement rates consist of unobservable hidden actions: the effect of longer sickness spells at the intensive margin and more sickness spells at the extensive margin.

We make two departures from the standard theoretical model, as outlined by Chetty (2006). On the cost side, we examine the relative contribution of the extensive and intensive margins by allowing the probability of becoming sick to vary in the model as a function of effort, which is unobservable to the social planner.^2^ On the benefit side, we allow for a fixed cost of sickness (θ in the model); that is, we depart from the standard assumption of the state independence of marginal utility.^3^

Given a concave utility function, the fixed cost of sickness, which fundamentally affects the utility gain of consumption smoothing, could, in principle, be of either sign. A positive (negative) fixed cost of sickness implies a positive (negative) state dependence, meaning that the marginal utility is higher (lower) in the sick state. The importance of state dependence in optimal sickness insurance has been acknowledged since at least the publication of the work of Zeckhauser (1970) and Arrow (1974). The state dependence implied by the presence of a fixed cost of sickness underlines the importance of interstate consumption smoothing of social insurance. Prior evidence (see Finkelstein et al. 2009, for a review), however, focuses on the relationship between health (e.g., Finkelstein et al. 2013, study of chronic disease) and marginal utility. Our focus is on the relationship between sickness absence and marginal utility. In what follows, we characterize and estimate θ.

The model yields an implicit equation for the optimal benefit, *b*, which is based on the sufficient statistics approach (an augmented Baily‐Chetty formula; see Supporting Information S1: Appendix 2 for a detailed derivation of the model):

(1) $${\epsilon_{r,b}+\epsilon }_{D,b}=\frac{u^{\prime }\left(c_{s},1\right)-u^{\prime }\left(c_{e},0\right)}{u^{\prime }\left(c_{e},0\right)}\approx \gamma \frac{\Delta c+\theta }{c_{e}}\left[1+\frac{1}{2}\rho \frac{\Delta c+\theta }{c_{e}}\right],$$

where $\epsilon_{r,b}=\frac{d\log\;\left(\frac{p}{1-p}\right)\;}{dlog\;\left(b\right)\;}$ is the elasticity of the odds ratio ($r=\frac{p}{1-p}$) of sickness leave with respect to the sickness benefit (i.e., the extensive margin); $\epsilon_{D,b}=\frac{dlog\;\left(D\right)\;}{dlog\;\left(b\right)\;}$ is the elasticity of the duration (*D*) of sick leave with respect to the sickness benefit (i.e., the intensive margin); $u\left(c_{e},0\right)$ and $u\left(c_{s},1\right)$ =  $u\left(c_{s}-\theta ,0\right)$ are the utility functions; $c_{e}$ and $c_{s}$ are consumption in the states of employment (S=0) and sickness leave (S=1), respectively; $\frac{\Delta c}{c_{e}}=\frac{c_{e}-c_{s}}{c_{e}}$ is the proportional drop in consumption when on sick leave; $\gamma =-\frac{{c_{e}u}^{\prime \prime }\left(c_{e}\right)}{u^{\prime }\left(c_{e}\right)}$ is the coefficient of relative risk aversion; and $\rho =-\frac{{c_{e}u}^{\prime \prime \prime }\left(c_{e}\right)}{u^{\prime \prime }\left(c_{e}\right)}$ is the coefficient of relative prudence.The envelope theorem guarantees that all other behavioral responses can be ignored when setting the optimal benefit level, except the elasticity parameters ($\epsilon_{D,b}$ and $\epsilon_{r,b}$) that enter the government budget constraint directly (Supporting Information S1: Appendix 2).

The model has an intuitive interpretation. The left‐hand side of the equality in Equation (1) disentangles the extensive ($\epsilon_{r,b}$) and intensive ($\epsilon_{D,b}$) margins of the effect due to hidden actions, that is, moral hazard. The right‐hand side of Equation (1) defines the value of the insurance (i.e., the change in relative marginal utility) under sick leave, which is affected by the state‐dependent nature of marginal utility, captured by θ. The reduction in consumption is a function of the replacement rate. We estimate the utility function parameters, including the fixed cost of sickness, while allowing the value of insurance to vary as a function of income (for more details, see Section 3).

The Baily‐Chetty formula is based on simplifying assumptions. The model does not account for any possible preference for vertical redistribution across individuals, general equilibrium effects on wages, the marginal cost of public funds, externalities on government budgets, or other externalities (Pichler and Ziebarth 2017). Moreover, reference dependence might play a role in the utility function in this context (Kahneman and Tversky 1979). However, any reference dependence not captured by θ is not considered.

## 3 Empirical Approach

### 3.1 Data

Our main dataset is the Socio‐Economic Panel (SOEP), which is a large survey of German households on a wide range of variables, including incomes and subjective well‐being.^4^ We restrict the data to the years 1993–2018; therefore, all our relevant variables are found for all the years.^5^

The level of life satisfaction is measured on an 11‐point scale from 0 to 10, where 0 is “not at all satisfied” and 10 is “completely satisfied.” We give the life satisfaction variable a cardinal (not ordinal) interpretation to accomplish our analyses, following, for example, Layard et al. (2008), who require comparability of utility across individuals.

There is an extensive literature debating happiness scales (see e.g., Bond and Lang 2019; Chen et al. 2019; Kaiser and Vendrik 2020). Kaiser and Oswald (2022) provide empirical evidence which shows that the relationship between categorical life satisfaction variables in different life domains and economic behavior “is fairly close to linear” at least in some contexts and that a single subjective measure predicts subsequent behavior better than a set of around 20 economic and social variables in most cases. Kaiser and Vendrik (2020) specify that in some non‐linear scale transformation cases estimates might be sensitive. We emphasize that our main parameter of interest, the fixed cost of sickness is essentially a horizontal shift parameter of the utility function in the sick state and should allow for a relatively stable estimation. Indeed, the set of specifications that we preform produce fairly stable estimates for the fixed cost of sickness with the exception of the specifications in which we switch the sickness variable to disability or measures of more severe sickness. In those cases, the changes in the estimates seem reasonable (see Supporting Information S1: Table A2). For sensitivity analysis, we run our main model with a binarized outcome variable (Supporting Information S1: Table A1 and Figure A4). One potential issue in using subjective measures of well‐being is heterogeneity in the way individuals use measurement scales, or scale use heterogeneity (see Benjamin et al. 2024). In our analysis, we mitigate concerns related to selection bias and unobserved heterogeneity, including scale use heterogeneity, by focusing on switchers, ensuring that our estimates capture meaningful within‐individual variation. However, within our sample of switchers, if those with a higher proportion of years in sickness use a different scale compared to those with fewer sickness years, this would bias our estimates. Unfortunately, we cannot infer the direction and size of the potential bias.

The measure of subjective well‐being is life satisfaction, as it is the best available proxy for decision utility (Benjamin et al. 2012, 2014; Benjamin, Heffetz, Kimball, and Szembrot 2014; for a discussion of decision vs. experienced utility, see Kahneman et al., 1997). Kimball and Willis (2023) argue clearly that happiness (or life satisfaction) is a distinct concept from utility and that it is a sum of elation, or short‐term happiness, and baseline mood, or long‐term happiness. Baseline mood is seen as (only) one argument in the utility function. This conceptual framework motivates the use of subjective well‐being measures but places them in a specific relationship to utility. Any changes in subjective well‐being will be informative of changes in utility. However, life satisfaction is affected by context and framing and we cannot overrule that sickness itself could act as a change of context. Thus, although life satisfaction is a useful empirical measure, it is limited in its ability to directly measure economic utility.

The income variable we use for all our analyses is net income. A year with sickness is defined by the question asking whether the individual spent more than 6 weeks in sickness absence in the previous year; we then assign a sickness designation to the relevant year. Using panel data enable us to accurately align reported periods of sickness absence with income data. Note that our variable does not allow us to separate the effect of sickness from that of sickness absence. We use both terms interchangeably. However, our focus is on the policy, which is conditional on sickness absence, regardless of the exact nature of the sickness. An individual might also not be sick during the interview. Our estimates should be interpreted as a wider and long‐term effect of sickness on life satisfaction. Cases in which the interview was held before the onset of the sickness imparted a measurement error and a resulting attenuation bias. For a more general interest, we also study the sensitivity of our results to the changes in the sickness variable from sickness absence to specific measures of sickness (see Supporting Information S1: Table A2).

We restrict our sample to year‐person observations in which the individual worked more than 1000 h at the ages 19–64 years. We construct our sample by selecting those who switch between the sickness and healthy states during the sample period. This focus on “switchers” ensures that the estimated utility curve for the sick and the healthy is estimated using the same individuals. We also show our results for differently constructed samples. Descriptive statistics are presented in Table 1. Supporting Information S1: Figures A1–A2 display the histograms of the incomes and life satisfaction of the two subsamples (sick leave vs. healthy), respectively. The number of year‐individual observations is larger (∼56,000) in the healthy state than in the subsample for those in the sick state (∼11,000), since the “switchers” have more healthy years than sick years. Overall, 6994 different individuals are included, with an average of 8.97 periods, of which 1.48 are in sickness. The mean life satisfaction is ∼0.3 points lower in the sick state.

**TABLE 1:** Descriptive statistics.

	Healthy		Sick	
	Mean	SD	Mean	SD
Life satisfaction	6.97	1.65	6.65	1.85
Net income	47,426.65	28,599.94	45,656.40	36,880.43
*N*	56,268	—	10,961	—

*Note:* The healthy state is defined as not having had a sickness absence of at least 6 weeks during the year. The sick state is defined as having had a sickness absence of at least 6 weeks during the year. There are 6994 different individuals with, on average, 8.97 periods of which 1.48 were in sickness.

### 3.2 A Utility Function Compliant With the Data

**FIGURE 2:**
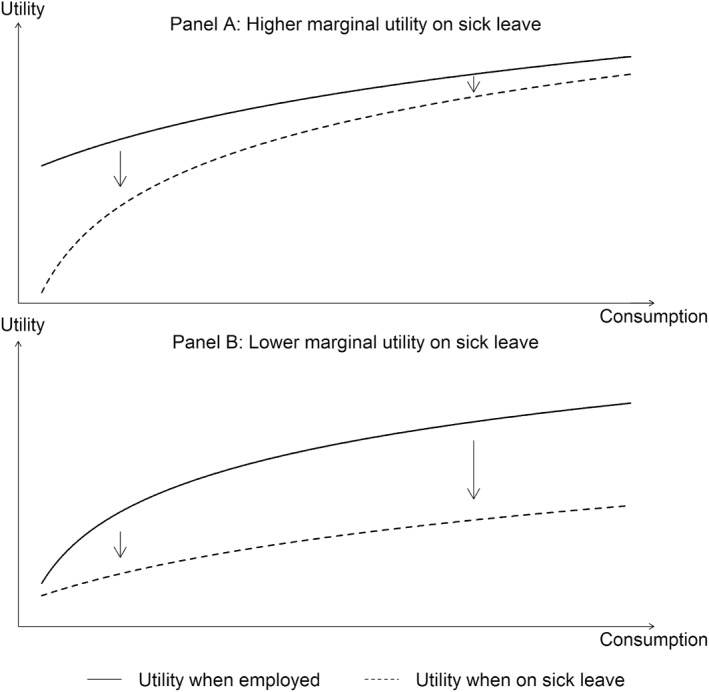
Conceptual framework describing marginal utilities. Adapted from Finkelstein et al. (2013). (Panel A) The panel presents a utility function with positive state dependence, that is, a utility function with higher marginal utility at each consumption level when on sick leave. (Panel B) The panel presents a utility function with negative state dependence, that is, a utility function with lower marginal utility at each consumption level when on sick leave.

We estimate the utility functions in the healthy and sick states using life satisfaction as a measure of subjective well‐being, allowing us to infer both the degree of risk aversion and state dependence. No consensus exists regarding the sign of state dependence, possibly due to varying contexts (see Finkelstein et al. 2009, 2013; Figure 2).

For a non‐parametric analysis of the relationship between income and life satisfaction in the data, we fit a spline. A visual inspection of the spline fit of the income–well‐being gradient, as shown in Figure 1, reveals a lower utility for persons on sick leave but with a higher slope, compared with those who are employed. To account for this relationship, we utilize a parametric functional form in the family of HARA (Hyperbolic Absolute Risk Aversion) utility functions. This offers a flexible and tractable functional form that encompasses the most commonly used functions in macroeconomics and finance and emerges from economic reasoning (Perets and Yashiv 2016). The HARA utility functions can account for an increasing, decreasing, or constant relative risk aversion (see Merton 1971; D. J. Meyer and Meyer 2005, for a review):

(2) $$u\left(c\left(y\right),S\right)=\frac{\gamma }{1-\gamma }{\left(\frac{\alpha_{H}y-\omega -\theta S}{\gamma }\right)}^{1-\gamma },$$

where S is an indicator for sickness leave and ω is a horizontal shift parameter. Similar shift parameters have been used in dynamic analyses incorporating stock effects, such as habit formation (see Phlips 1978). As in the economic model presented in Section 2, θ is the fixed cost of sickness. For simplicity, we set the income scale parameter $\alpha_{H}=1$ (see Section 3.3. for more details). In applying the model to life satisfaction data, we augment Equation (2) with parameters accounting for the scale used to measure life satisfaction and a vertical shift parameter in mean level of life satisfaction in the sick state. Including these parameters do not affect the formula for the relative marginal utilities in Equation (1), nor the values of relative risk‐aversion, see Equation (6) below. The vertical shift parameter gives flexibility in allowing for both a lower and a higher marginal utility and utility level in the sick state.

Unlike in the standard CRRA (Constant Relative Risk Aversion) model, the relative risk aversion (RRA) and relative prudence (RP) are functions of *y* and ω. Assuming that agents also know their utility function in the sick state, their relative risk aversion and relative prudence are also functions of θ:

(3) $$RRA_{S}\left(y,1\right)=-y\frac{u^{\prime \prime }\left(y,1\right)}{u^{\prime }\left(y,1\right)}=\frac{\gamma y}{y-\omega -\theta },if\;c>\omega +\theta$$

(4) $$RP_{S}\left(y,1\right)=-y\frac{u^{\prime \prime \prime }\left(y,1\right)}{u^{\prime \prime }\left(y,1\right)}=\frac{\left(\gamma +1\right)y}{y-\omega -\theta },if\;c>\omega +\theta .$$

Using the functional form from Equation (2) and assuming that net income is a near equivalent to consumption (but see equation (13)), we can explicitly solve Equation (1) for the optimal replacement rate (RR):

(5) $$RR=1-\frac{\Delta y}{y_{e}}=\left(\frac{\omega }{y_{e}}+\frac{\theta }{y_{e}}\right)+{\left(1-\frac{\omega }{y_{e}}\right)\left(1+{\epsilon_{r,b}+\epsilon }_{D,b}\right)}^{-\frac{1}{\gamma }},$$

where $\Delta y=y_{e}-y_{s}$. Equation (5) yields the optimal benefit schedule in terms of replacement rates, conditional on the income level. Note that the presence of θ on the right‐hand side of Equation (5) implies that both pecuniary and non‐pecuniary costs must be considered when characterizing the total benefit of insurance. If θ>0, the state dependence is positive and vice versa.

### 3.3 Estimation and Identification

For the empirical specification of the utility function, we estimate a non‐linear least squares fit of the following form:

(6) $$SWB\left(c\left(y_{i}\right),S_{i}\right)=\alpha +\frac{\beta }{1-\gamma }{\left(\frac{y_{i}-\omega -\theta S_{i}}{\gamma }\right)}^{1-\gamma }+\delta S_{i}+{\tau X}_{i}+\varepsilon_{S_{i},i},$$

which is Equation (2) augmented with additional parameters (the constant α and the level effect of sickness absence δ) to increase flexibility. $S_{i}$ is the sickness leave indicator, $y_{i}$ is net income, and $SWB_{i}$ is life satisfaction. The sick state ($S_{i}=1$) is measured as the state of having sickness absences for more than 6 weeks during the year.^6^ The healthy state ($S_{i}=0$) is measured as spending fewer than 6 weeks in sickness absence (see Section 3.1. for more details).^7^

Each parameter in the non‐linear component $\frac{\beta }{1-\gamma }{\left(\frac{y_{i}-\omega -\theta S_{i}}{\gamma }\right)}^{1-\gamma }$ of Equation (2) defines the form and location of the utility curve. The fixed cost of sickness, θ, gives the horizontal shift to the curve in the sick state, and its sign defines whether the marginal utility in the sick state is higher or lower than in the healthy state. Empirically, we observe both negative and positive values of θ, underlining the flexibility of the specification (see Supporting Information S1: Table A2). The parameter ω gives the horizontal shift of the curve in both states. The γ parameter is the traditional measure for risk aversion, that is, it affects the curvature of the utility curve, conversely, the curvature of the data identifies this parameter. However, due to the presence of the ω and θ parameters, relative risk aversion is a function of income. See Section 4.2 and Supporting Information S1: Figure A3 for more details. The parameters α (constant) and β (scale parameter), whose values are not our focus, are included to account for the scale used to measure life satisfaction. Note that parameters α, β, and the level effect of sickness absence, δ, do not affect the relative marginal utility in Equation (1) nor the value of relative risk aversion.

The ω parameter is crucial in fitting the level of well‐being for low‐income individuals. In essence, assuming that all utility is a function of consumption, it plays a major role in defining the level of utility at a very low income. At zero income, savings can be consumed, but it also publicly provides goods and services. We consequently call this parameter the “institutions parameter”. We recognize that much more goes into this parameter than only publicly provided goods and services, such as access to credit and the functioning of financial markets. To better understand its nature, we implement a multi‐country analysis using EU‐SILC data. We find that the highest country correlation with the “institutions parameter” is with trust in institutions (ρ=0.82) and interpersonal trust (ρ=0.80). For a more direct measure of the magnitude of the welfare state (i.e., social spending in euros) yields a lower correlation of 0.49 with the institutions parameter. (See Supporting Information S1: Appendix 5 for EU‐SILC aggregate analysis and Supporting Information S1: Appendix 6 for EU‐SILC country‐level analysis, which also includes the above‐mentioned correlations.) Finally, we also include a set of controls, X: age, age squared, household size, female dummy, and whether the individual is single.

We apply the R package “minpack‐lm”, which is based on a modified Levenberg‐Marquardt‐type algorithm to obtain our fit. The maximizing problem is non‐smooth across the boundary of having the value one in the γ parameter. We choose the fit with the lowest sum of squared errors across the regions, which is obtained with an above‐one initial γ parameter value.

If γ>1, Equation (6) yields a bounded utility function. Given the state‐dependent shift and level parameter, δ, the model can flexibly fit both positive and negative state dependence according to the data. For example, a relationship shown in Panel B of Supporting Information S1: Figure A3 would be produced by θ<0 and δ<0.

The functional form (6) corresponds to HARA $\left(U_{HARA}\left(c\left(y\right)\right)={\frac{\gamma }{1-\gamma }\left(\frac{\alpha_{H}y+\beta_{H}}{\gamma }\right)}^{1-\gamma }\right)$, with the simplifying restriction that $\frac{\alpha_{H}}{\gamma }=\frac{1}{1000}$ (i.e., we measure income in thousands of annual euros). Scaling income to a similar order of magnitude as life satisfaction slightly increases estimation robustness due to particulars of the numeric estimation algorithm. The numerical values of the parameters of interest {θ,ω} remain stable but are scaled by $\frac{1}{1000}$, and the estimation of the crucial parameter γ is not qualitatively affected by the scale.

Empirically, at any point in time, we assume that a difference exists in the marginal utility of the healthy (S=0) and sick (S=1) populations and is captured by the fixed cost of sickness (θ). The parameter θ can be decomposed into two parts, $\theta =\theta_{s}+\theta_{b}$, where $\theta_{s}$ is the effect of sickness absence across states and $\theta_{b}$ is the difference in the utility across individuals. The latter component is the selection or estimation bias. Since we estimate θ using the same individuals in the two states, $\theta_{b}$ is likely to be small. For the policy to be pure insurance, the social planner will only consider $\theta_{s}$. To maximize utility across states and across individuals, the social planner will consider both components.

The correct interpretation of the fixed cost of sickness is the loss of utility in monetary terms as a direct result of sickness. Any income change due to sickness will also affect utility. The difference in net incomes is only around 4% between the sick and healthy years; thus, the effect of changed incomes is bound to be small (Table 1). Our strategy of using the switcher population is also aimed at minimizing selection bias and, thus, at correctly identifying the fixed cost of sickness. However, since we do not employ individual fixed effects in the estimation, there is a potential concern about omitted variable bias. The numerical method that we use and the nonlinear specification do not allow us to include individual fixed effects. In our empirical application, the use of individual fixed effects based on within‐individual variation over time could also increase the measurement error in self‐reported variables and attenuate the parameter estimates. We focus on the fixed cost of sickness, and for our interpretations, we assume that an independent measurement error is present between the fixed cost of sickness and the outcome variable.

## 4 Results

### 4.1 Estimation Results

To model the empirical relationship observed in Figure 1 and to obtain the numerical estimates of the parameters of the utility function, including the fixed cost of sickness, we fit Equation (6). The estimated parameter values are documented in Table 2. The main specification result is presented in column 1 of Table 2. Of the parameters with policy significance, {γ,θ,ω}, the first two are statistically highly significant. The fixed cost of sickness is estimated to be approximately five thousand euros per year. There is also a statistically significant drop in the estimated utility curve of 0.21 life satisfaction points for those with sickness.

**TABLE 2:** Main estimates.

	Main specification	Years 1992–2004	Years 2005–2018	Years 2015–2018	Main specification without controls
	(1)	(2)	(3)	(4)	(5)
Constant (α)	9.93*** (0.20)	10.52*** (0.64)	9.35*** (0.22)	9.69*** (0.39)	7.75*** (0.17)
Scale parameter (β,thousands)	2.79 (14.21)	1.37 (16.66)	9.03 (66.73)	0.07 (0.28)	2.10 (11.01)
Relative risk aversion parameter (γ)	3.19** (1.39)	2.95 (3.33)	3.71* (2.15)	2.38** (1.18)	3.09** (1.41)
Institutions parameter (ω)	−56.49 (39.35)	−51.85 (91.74)	−54.53 (50.28)	−24.04 (25.17)	−57.95 (41.91)
Fixed cost of sickness (θ)	4.86*** (1.31)	2.51 (2.25)	4.44*** (1.54)	8.22*** (3.11)	6.28*** (1.50)
Level effect of sickness (δ)	−0.21*** (0.04)	−0.16*** (0.08)	−0.24*** (0.04)	−0.09 (0.07)	−0.17*** (0.04)
Individual controls	Yes	Yes	Yes	Yes	No
Year fixed effects	Yes	Yes	Yes	Yes	No
*N*	67,229	19,395	32,423	9302	67,229

*Note:* Statistical significance: * *p* < 0.1; **p < 0.05; ***p < 0.01. The non‐linear regression is the fit using a modified Levenberg‐Marquardt‐type algorithm with sampling weights. The standard errors are in parentheses. All models are estimated with Equation (6). Individual controls are age, age squared, household size, female dummy, and a dummy for being married. The starting values, where applicable, are: {α=10,β=0,γ=2.7,ω=−15,θ=15,δ=0} and zero for all other parameters. For the estimation, the income variable is in thousands of annual euros. The population is limited to “switchers” (i.e., those who had one or more years with sick leave and one or more years without sick leave). To be defined as having sick leave, a person requires 6 weeks of absences. In columns 2 and 3, we replicate the main specification for years before and after the start of 2005, respectively. In column 4, we exclude years when an individual switches between sickness states. In column 5, we include only those years when an individual switches between sickness states.

**FIGURE 3:**
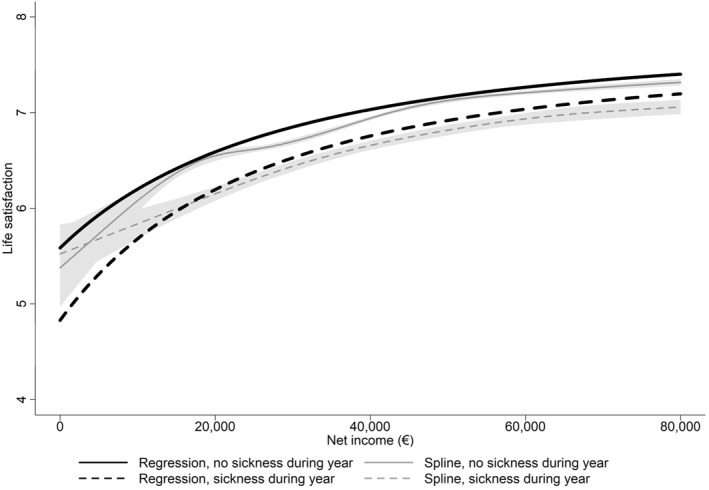
Spline and non‐linear fit of life satisfaction and net income split by sickness states. The non‐parametric estimate is a spline fit. The x‐axis in the figure is truncated at 80,000 euros. The gray area around the curves represents the 95% confidence interval. The non‐linear regression in the black is from equation (6), fitted values in Table 2, model 6. Sample size: 56,268 years‐person observations with no sickness during the year, 10,961 years‐person observations with sickness during the year.

The utility curves, overlaid on the spline fit, are presented in Figure 3. The fit in the graph is done without controls (Table 2, column 6). The estimates confirm the visual observation of a steeper association between life satisfaction and income, conditional on income in the sickness absence state. Using our functional form, the positive state dependence stems from the positive and significant fixed cost of the sickness parameter, θ. Visually, the fitted curve describes the spline fit rather well, except at low incomes below 5000 to 10,000 euros. Net incomes below 10,000 euros represent only 0.8% of the sample. The fitted utility curves, unlike the spline curves, are weighted by sample weights. Comparing columns 1 and 6 in Table 2 suggests that the key parameters of the estimated HARA utility curve are not sensitive to controls.

In columns 2 and 3, we split the sample at year 2005 and compare the period 1992–2004 to 2005–2018. The fixed cost of sickness is insignificant for the former and significant and similar to the whole sample estimate for the latter. In column 4, we use only the last 4 years of the sample (2015–2018). We find a large and positive fixed cost of sickness.

Across all samples, the estimated relative risk aversion parameter (γ) is relatively stable, varying between 2.95 and 3.71 in the point estimate and making the institutions parameter crucial in defining the form of the curve.

We replicated all our results with the EU‐SILC cross‐sectional data for European countries. The results are presented in Supporting Information S1: Appendices 4 and 5. Further sensitivity analyses are presented in Section 4.3.

### 4.2 Policy Implications

We assessed the significance of our estimates to policy by taking the main specification results (Table 2, column 1) and exploiting the optimal policy that the Bailly‐Chetty model implies (Equation 1). First, we calculated the relative risk aversion (RRA) and relative prudence parameters using Equations (3) and (4). The former is shown in Supporting Information S1: Figure A3. The estimated RRA increases with income. Intriguingly, relative risk aversion is higher in the sick state, consistent with the finding of Decker and Schmitz (2016) that self‐reported risk aversion increases after health shocks. This result stems from the −ω and −θ terms in the denominator of Equation (3). As long as −ω−θ>0, RRA will increase and converge toward γ as incomes increase. In essence, the pattern of increasing RRA follows from having a negative horizontal shift parameter or institutions parameter ω. Intuitively, lower‐income individuals have smaller risks, since they are relatively more protected by the “institutions.” This result challenges the conventional wisdom of decreasing relative risk aversion (see D. J. Meyer and Meyer 2005). In our application, the emphasis is on the estimate for the sick state in which relative risk aversion is higher.

**FIGURE 4:**
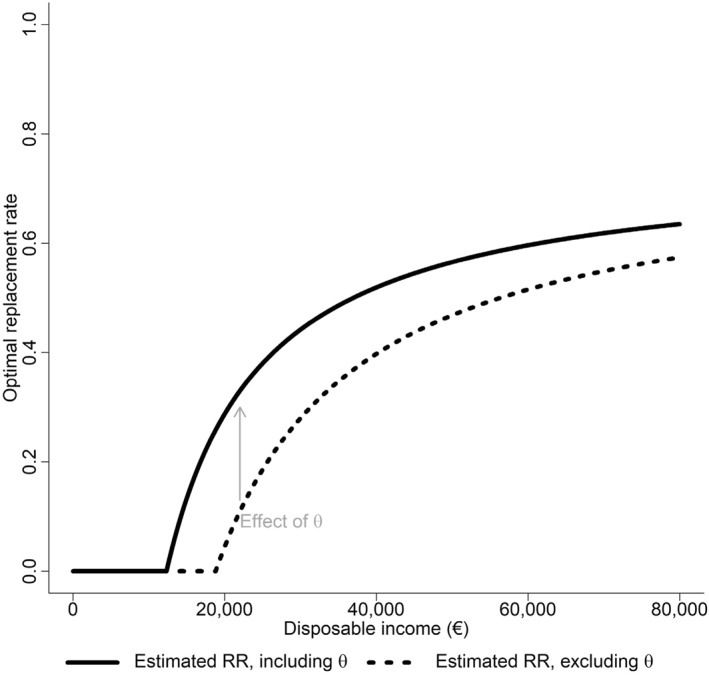
Optimal replacement rates. The optimal replacement rates are calculated using Equation (5). The relative risk aversion values are from a HARA utility function with the parameter values of {γ,ω,θ}={3.19,−56.49,4.86} at different levels of net income, shown in Table 2, model 1, obtained from estimating Equation (6). θ is the fixed cost of sickness, which affects the optimal replacement rate through relative risk aversion (RRA) and the augmented Baily‐Chetty formula. Additionally, we assume that ${\epsilon_{r,b}+\epsilon }_{D,b}=1.5$.

**FIGURE 5:**
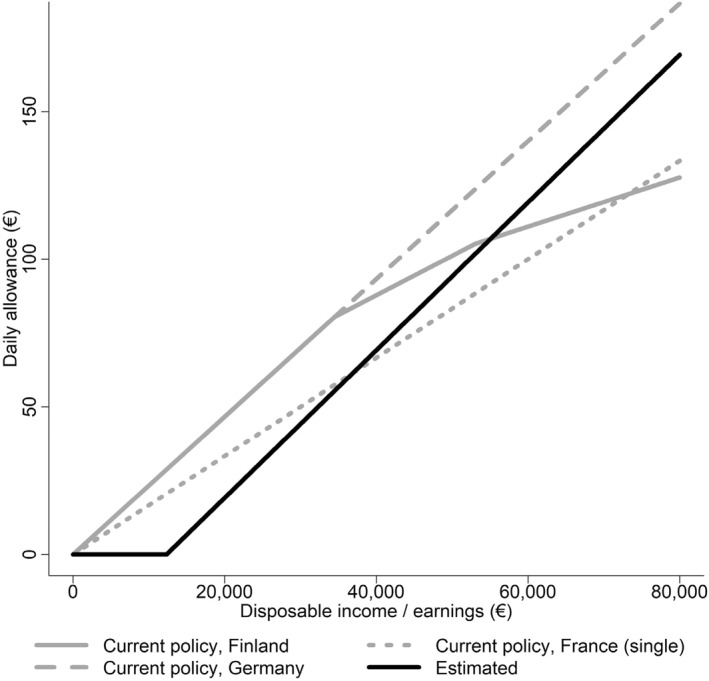
Prevailing universal sickness insurance policy and estimated optimal curves. The optimal replacement rates are calculated using Equation (5). The relative risk aversion values are from a HARA utility function with the parameter values of {γ,ω,θ}={3.19,−56.49,4.86} for the solid line at different levels of net income, shown in Table 2, model 1, obtained from estimating Equation (6). θ is the fixed cost of sickness, which affects the optimal replacement rate through relative risk aversion (RRA) and the augmented Baily‐Chetty formula. Additionally, we assume that ${\epsilon_{r,b}+\epsilon }_{D,b}=1.5$. “Single” refers to a one‐member household. The optimal curve is based on net income, whereas the current policy curves are based on earnings.

We apply Equation (5) to characterize the potential implications for the optimal policy of the estimated and assumed parameter values. We are also interested in the role of θ in determining the optimal policy curve (Figures 4, 5). Since the utility curve itself is estimated with different individuals across the income spectrum, we focus on the effect of the estimated fixed cost of sickness on optimal policy. We further assume that ${\epsilon_{r,b}+\epsilon }_{D,b}=1.5$, that is, the combined effect of the extensive and intensive margins sums to 1.5.^8^

We find that the optimal replacement rate curve is nonlinear and increases slightly with income. In Figure 4, we show that the fixed cost of sickness reshapes the replacement rate curve at the lower end of the income scale. For lower‐income individuals, who have some safety given by the horizontal shift parameter (ω), the effect of the fixed cost of sickness, or the state dependence of relative risk aversion, is the main reason to insure against sickness. A key feature is the higher relative risk aversion in the sick state (Supporting Information S1: Figure A3).

Figure 5 shows the optimal policy curve. Our estimated replacement rates for most income earners fall between the current French policy of a 0.5 replacement rate and the German policy of a 0.7 replacement rate. Note, however, that the optimal curve is based on net income, whereas the current policy schemes are based on earnings. Since our estimate for the utility curve itself is biased by the fact that it is estimated for different individuals across the income spectrum, the derived level of optimal policy is biased. However, the core observation arising from our estimates is that the utility function itself is different in the sick state due to a fixed cost of sickness. This state dependence has a significant effect on optimal policy, as shown in Figure 5.

### 4.3 Sensitivity Analysis

#### 4.3.1 Comparison to Previous Estimates

In this section, we compare our results to previous estimates, placing special attention on the work by Finkelstein et al. (2013). Using U.S. panel data, Finkelstein et al. (2013) estimate that an increase of one standard deviation in the incidence of chronic disease leads to a 10%–25% drop in the marginal utility of consumption relative to the healthy population. Their result is thus the opposite of ours.^9^

Several possible reasons can explain this deviation. First, the main specification by Finkelstein et al. (2013) is a log‐linear mapping from income to well‐being; that is, they assume a relative risk aversion of 1, although they find that their result is robust to some relaxation of this assumption. Conversely, we focus on a flexible nonlinear specification in which we estimate relative risk aversion (which is non‐constant by income), the level effect of sickness, and the fixed cost of sickness. Thus, their functional form is a special case of the one we utilize. We discuss the γ parameter in Section 3.4.2, including the log‐linear functional form.

Second, their response variable is a binary happiness indicator, as opposed to our cardinal measure of life satisfaction. We study the robustness of our main result to the binarization of the outcome variable at 4 different cutoff points (6–9) in the 11‐point life satisfaction measure. In Supporting Information S1: Table A1 and Figure A4, we show the results of this binarization. As the table reveals, in most binarizations, we lose the statistical significance of the estimate for the fixed cost of sickness. The choice between the cardinal interpretation of life satisfaction as in our main specification and binarization is not inconsequential. Supporting Information S1: Table A1 also shows that the choice of the cutoff point has a meaningful effect on results. In the first column, in which the cutoff point is at six, the point estimates for the relative risk aversion, institutions and the fixed cost of sickness parameters are similar to our cardinal main specification. The standard error for fixed cost of sickness—our main parameter of interest—in the first column is also the lowest. It seems that a lot of the relevant variation in our non‐linear context is in the middle of the scale. Finkelstein et al. (2013) use a binary survey question. In their sample, 87% of respondents answer that they were happy “much of the time during the past week”. This proportion corresponds closest to the proportion at cutoff 6 (Supporting Information S1: Table A1, column 1). The results in Supporting Information S1: Table A1 also call for caution when choosing the binarization cutoff point.

Third, Finkelstein et al. (2013) study the effect of chronic conditions, whereas we examine any sickness absence of above 6 weeks in length. In columns 1 to 6 of Supporting Information S1: Table A2, we vary the definition of sickness. First, we interact sickness absence with also having at least 8 doctor visits per year. Our sample size drops steeply, since we only include “switchers,” or those who have both sickness and healthy years. The points estimate for the fixed cost of sickness also drops and is no longer significant, partly due to larger standard errors. In the next three columns, we no longer study the effect of sickness absence. Instead, to align closer to the specification of Finkelstein et al. (2013), we define sickness by the number of doctor visits alone. Individuals with at least 8 doctor visits show no significant fixed cost of sickness. With at least 28 and 48 doctor visits, we now observe a large positive fixed cost of sickness, even with the smaller sample sizes. In column 5, we look at sickness absences again, but we now only include sickness absences of at least 180 days. The benefit of focusing on this population is that each individual has at least a 50% probability of being on sick leave when the interview is conducted. This focus reduces the sample size substantially to less than one‐10th of our original sample. We drop our control variables to allow a sensible fit of the model. The results show a similar and statistically significant fixed cost of sickness, as we observe for our main specification. The sixth column, however, reports the results for disability as the sickness variable, not conditioned on sickness absence. This definition is close to that of Finkelstein et al. (2013) and indeed yields a negative and significant estimate for the fixed cost of sickness. Supporting Information S1: Figure A5 confirms graphically the implied negative state dependence as conceptualized in Figure 2, Panel B. The estimate for the level shift in life satisfaction is also negative and significant, highlighting the flexibility of our specification. We observe a lower marginal utility and utility levels for the disabled state. This is consistent with the concept of a lowered capability to derive utility from a given level of income when disabled. We also report the disability specification without controls (column 7) for reference when we compare different samples below. The estimates are similar both with and without controls (columns 6 and 7).

Fourth, the two studies focus on contrasting populations. Finkelstein et al. (2013) studied persons aged 50 years and above who are not in the labor force. We studied the working‐age employed population. In Supporting Information S1: Table A2, column 8, we replicate column 7, but now focus on a population aged 50 years and older. Here again, we employ a specification without controls since the small sample size of around 10,000 observations does not allow for the proper use of control variables. The estimate for the fixed cost of sickness remains negative, although not statistically significant. Finally, in column 9, we replicate our main specification with the older sample, dropping the age‐squared variable due to having few age groups, and we obtain a result that is generally in accordance with our main specification. However, in this specification, the fixed cost of sickness is close to zero and not significant.

Comparing all the differences in our specifications and the methods we study them, the type of sickness appears to be pivotal for the sign of the fixed cost of sickness. Notably, disability seems to lower both the utility levels and marginal utility. In this paper, our focus is on sickness insurance; therefore, we are primarily interested in overall sickness absence. We are not able to study the interaction between sickness absence and disability due to the very small sample size.

To further understand the relationship between our results and the ones in Finkelstein et al. (2013), we re‐estimate all our specifications based on a loglinear relationship between life satisfaction and net income. This simplified form of the Finkelstein et al. (2013) approach allows us a straightforward use of individual fixed effects. The estimated model has the following structure:

(7) $$LS_{\text{it}}=\lambda_{i}+\gamma_{t}+\beta_{1}Y_{\text{it}}+\beta_{2}\text{Sic}k_{\text{it}}+\beta_{3}Y_{\text{it}}*\text{Sic}k_{\text{it}}+X_{\text{it}}^{\prime }\delta +\epsilon_{\text{it}},$$

where $LS_{\text{it}}$ is life satisfaction for individual i at time t, $\lambda_{i}$ and $\gamma_{t}$ are the individual and time fixed effects, $Y_{\text{it}}$ is log net income and $X_{\text{it}}^{\prime }$ is a vector of controls. Individual controls are—as before—age, age squared, household size, female dummy, and a dummy for being married. The results are reported in Supporting Information S1: Appendix 1.2. In interpreting these results, we highlight a concern about using individual fixed effects with subjective well‐being measures. Utilizing individual fixed effects, which are based on within‐person variation over time, might amplify the measurement error in self‐reported variables and diminish the accuracy of parameter estimates.

Looking at all the replication Supporting Information S1: Tables A1.2.1., A1.2.2. and A1.2.2. (replicating Table 2, Supporting Information S1: Table A1 and A2, respectively), we find significant estimates for the interaction of log net income and sickness. All significant estimates are negative, supporting Finkelstein et al. (2013) findings. The first column of Supporting Information S1: Table A1.2.1. shows that the point estimate of the parameter of interest is non‐significant and negative. Simply using a different specification, one close to the type of Finkelstein et al. (2013), gives a result with a negative effect of sickness on marginal utility. However, the estimates vary rather strongly in columns 2 to 4, which employ different year ranges. There seems to be an upward time‐trend in the estimate similarly to our main model. In column 5, for completeness, we show the estimates with no controls and find an insignificant positive estimate for the interaction. Column 6 replicates column 1 but without individual fixed effects. The parameter estimate for the interaction of log income and sickness does not change much when dropping individual fixed effects. In this context we do not find a large importance for the individual fixed effects regarding the estimate for the parameter of interest. This is reassuring, since we are not able to include individual fixed effects in our main model due to its non‐linearity and numerical estimation.

In Supporting Information S1: Table A1.2.2., we estimate the models with binarized outcomes. First, when life satisfaction is expressed as one for the values of 8 and above and zero otherwise, we find a different slope in the sick state compared to the healthy state. The other three binarized outcome regressions yield insignificant estimates, which again raises the concern about the choice of the correct cutoff point. In Supporting Information S1: Table A1.2.3. we observe that again disability as the outcome variable yields the largest negative effect on the slope in the sick state, as in the main model. Also, using more severe definitions of sickness (at least 28 annual doctor visits, at least 48 annual doctor visits and sickness absence of at least 180 days) yield insignificant positive effects on the marginal utility of sickness, similarly to the main model in which the point estimates for these three variables were larger than the positive effect found in the main specification.

We have thus acquired two sets of estimates for the effect of sickness on the income‐well‐being gradient. One set is based on our main model, which has the benefits of estimating the monetary value of sickness and having more statistical power in detecting the effect. The other set is from the loglinear fit, which has the advantage of adding individual fixed effects. The downside of our model is the lack of individual fixed effects, whereas the loglinear fit does not have the precision of the main model and it does not offer as good a fit when we compare it to our model without individual fixed effects. We have estimated in all 18 common specifications of these two models.

We plot the estimates for the effect of sickness on marginal utility from both models in Supporting Information S1: Figure A1.2.1. Black dots depict different samples (Tables 2 and Supporting Information S1: A1.2.1), red dots represent the results from binarization (Supporting Information S1: Tables A1 and A1.2.2), and blue dots show the estimates for different sickness definitions (Supporting Information S1: Tables A2 and A1.2.3). Statistical significance at the 10% level is depicted by horizontal lines below the dots for the main model (x‐axis) and vertical lines on the left‐hand side of the dots for the loglinear fit (y‐axis). Only two of the specifications are significant for both models. Disability as the sickness variable leads to a significant negative estimate in both models (Supporting Information S1: Tables A2 and A1.2.3, column 6). The estimates for years 2015–2018, on the other hand, are positive (at the 10% level) in both models (Tables 2 and Supporting Information S1: A1.2.1, column 4). The red dots show that although it is a different binarized specification that is significant in both models, the estimates of the two models seem to behave similarly as a function of the choice of cutoff point. When we run a regression of loglinear estimates on the main model estimates, we find a statistically insignificant intercept of −0.015, suggesting that the loglinear model systematically yields more negative estimates for the effect of sickness on marginal utility than the main model. This result is visually confirmed by most of the 18 estimate pairs being below the 45‐degree line in Supporting Information S1: Figure A1.2.1. The correlation between the estimates is 0.59 (*p* = 0.01).

Supporting Information S1: Figure A1.2.2 plots the loglinear fit from Supporting Information S1: Table A1.2.1 against the spline fit. The figure looks very similar to Figure 3, except at low incomes, where the loglinear fit clearly fails to describe adequately the data. Using 6 parameters against the 4 in the loglinear fit allows for a visually better fit over the whole income distribution.

Relatedly, Viscusi and Evans (1990) estimate an optimal replacement rate of 0.85 using the U.S. data. However, the analysis by Viscusi and Evans (1990) is based on a relatively narrow survey dataset covering a group of U.S. chemical workers. By contrast, our data are nationally representative and cover the current German population. For this reason, and because substantial institutional differences are evident between the U.S. and Germany, the comparison of the estimates is not straightforward.

#### 4.3.2 Other Sensitivity Analyses

We perform a range of sensitivity tests for our main results. In the previous section, we studied the effect of (1) binarization of the outcome variable, (2) changing the definition of sickness, and (3) changing the population. We further study “adaptation” (i.e., the effect of adding the previous year's life satisfaction value as a control) (Supporting Information S1: Table A2, column 10). We find that the past value is indeed a highly significant predictor of current values of life satisfaction. In addition, the fixed cost of sickness is lower and not statistically significant.

We then move on to study the robustness of the estimation methodology to the starting values. Supporting Information S1: Figure A6, panels A to E, shows the theoretically important parameter values for 51 different starting values in those parameters, varying one parameter at a time for our main specification. The estimates are highly stable for alternative starting values for all the parameters except for γ. The least stable parameter is ω, which can be expected, since the standard errors are also very high. However, Panel B shows that when we vary starting values of γ, more variation appears in the estimates. Roughly, this variation can be grouped into three regimes: γ values below 1, γ values of 1–3.5, and γ values above 3.5. Given what we know about risk aversion, the two extreme regimes seem unlikely. First remember that our maximizing problem is non‐smooth across the boundary, γ=1. Therefore starting from below the value one does not guarantee that the global maximum is reached. This shows up as erratic behavior, if γ tends to 1. Also, at γ values above 3.5, the ω parameter behaves in an unstable manner. However, we do check the results for the three regimes side by side in Supporting Information S1: Table A3. The last row shows the residual standard errors, which are the lowest in our main specification and give the best fit by this standard. A very low or very high starting value in γ also results in a very high or a very low final estimate of γ. These two facts, in combination, lead us to conclude that a starting value of γ=2.7 is justified. For completeness, we also estimate the logarithmic function, following Finkelstein et al. (2013), implying that γ=1. The results are shown in column 4 of Supporting Information S1: Table A3. Again, the residual standard errors are higher than in our main specification, and other estimates differ considerably.

Overall, the conclusions of the sensitivity analyses of our empirical approach are: First, that a large sample size is needed for a proper estimation of the fixed cost of sickness. Second, the sign of the fixed cost of sickness also reverses when disability is studied rather than sickness absence, implying that different health conditions have opposing effects on marginal utility.

## 5 Conclusions

We studied the income–well‐being gradient in sickness and health to understand its state‐dependent nature. We extended the literature by recasting both the theoretical and the empirical methodology to account for state‐dependence in a tractable manner via the fixed cost of sickness term. We then show that the fixed cost of sickness is meaningful for optimal policy rules.

We obtained three main results focusing on sickness absence. First, the marginal utility is higher in the sick state, conditional on income (i.e., positive state dependence). We modeled this feature with a fixed cost of sickness. Second, relative risk aversion increases with income, stemming from the baseline security provided by “institutions” at lower incomes. Third, for lower‐income individuals, the main benefit from sickness insurance comes from insuring against the domain‐specific fixed cost of sickness. This result highlights the importance of domain‐specificity in optimal insurance design.

We propose the following procedure when assessing state‐dependent risk: First, estimate the standard measure of relative risk aversion in the state in which risk has not been realized. Second, evaluate the state dependence of marginal utility.

To identify utility functions, we assume that life satisfaction is a sufficiently satisfactory measure of an important dimension of utility, and that our fit of a flexible specification (Equation 6) guarantees a sufficiently good fit of the income‐well‐being gradient. We compare the same individuals in the two states. However, the baseline form of the utility curve is biased by comparing different individuals across the income distribution. Consequently, optimal levels of sickness insurance cannot be inferred from our work alone.

We thoroughly test our flexible model against an individual fixed effects loglinear model. The more flexible and precise main specification model teases out a significant state dependency in most specifications and provides a meaningful economic interpretation to the relevant estimates. The loglinear model is less precise in identifying a significant state dependency in most specifications. Both approaches consistently find a negative state dependency for disability. The loglinear fit replicates the finding that binarization of life satisfaction at different cutoff points gives different results. Empirically, it is not clear what cutoff point should be chosen if this route is taken. If the binarization approach is used, the robustness to different cutoff points should be shown.

The state dependence of the utility function implies that each relevant policy domain must be studied separately for optimal policy design. Future research should consider the role of fixed cost of other risks, such as unemployment and old age.

## Conflicts of Interest

The authors declare no conflicts of interest.

## Supporting information


Supporting Information S1

## Data Availability

The data that support the findings of this study are available on request from the corresponding author. The data are not publicly available due to privacy or ethical restrictions.

## Endnotes

^1^
See Palme and Persson (2019) for the description of sickness insurance systems in Europe.

^2^
This extension is helpful for our optimal policy discussion. Jäger et al. (2018) and Kolsrud et al. (2018), among others, study the extensive margin in the context of unemployment insurance.

^3^
See Chetty and Finkelstein (2013, 155–156) for an alternative way to incorporate state dependence.

^4^
Germany has an extensive and universal health care coverage. In addition, Germany has a universal sickness insurance for income protection, which is our focus. In Appendix 3, we describe briefly the sickness insurance system in Germany. In Supporting Information S1: Appendix 5.1, we compare sickness insurance systems in Europe, including Germany.

^5^
See https://www.diw.de/en/diw_01.c.678568.en/research_data_center_soep.html. The data are public and available to other researchers, and our method is replicable for sickness insurance and other policy domains.

^6^
We emphasize that our analysis aims to estimate the central parameters related to sickness insurance as they are practically implemented. Consequently, we focus on sick absence in our main specification, not specific types of sickness. The systems that have been implemented in Europe and elsewhere do not condition compensation schemes based on the nature of illness. See Section 3.3. for sensitivity analyses on the type of illness.

^7^
We measure income, not consumption. We discuss this distinction in Supporting Information S1: Appendix 4.

^8^
Echoing the estimate that we use in the calculations, Ziebarth and Karlsson (2014) argue that the consensus estimate of the literature is ∼1. Böckerman et al. (2018) also find that D,*b* ˜ 1.

^9^
In our estimation, we use the same set of controls as Finkelstein et al. (2013), except for individual fixed effects. This is a notable weakness of the numerical method that we apply in the paper. However, the use of fixed individual effects based on within‐individual variation over time may reinforce the potential measurement error in the self‐reported variables that attenuates the estimated coefficients.
