# Supplementary material for: Income–Well‐Being Gradient in Sickness and Health
Source: Health Econ. 2025 Nov 19;35(3):409–22. doi: 10.1002/hec.70063 (PMC12862129; doi:10.1002/hec.70063)
Supplement: Supplementary file 1 — Supporting Information S1 [file HEC-35-409-s001.docx]

ONLINE SUPPLEMENTARY APPENDIX

**Appendix 1: Additional figures and tables**

Figure A1. Density of incomes by sickness status

*
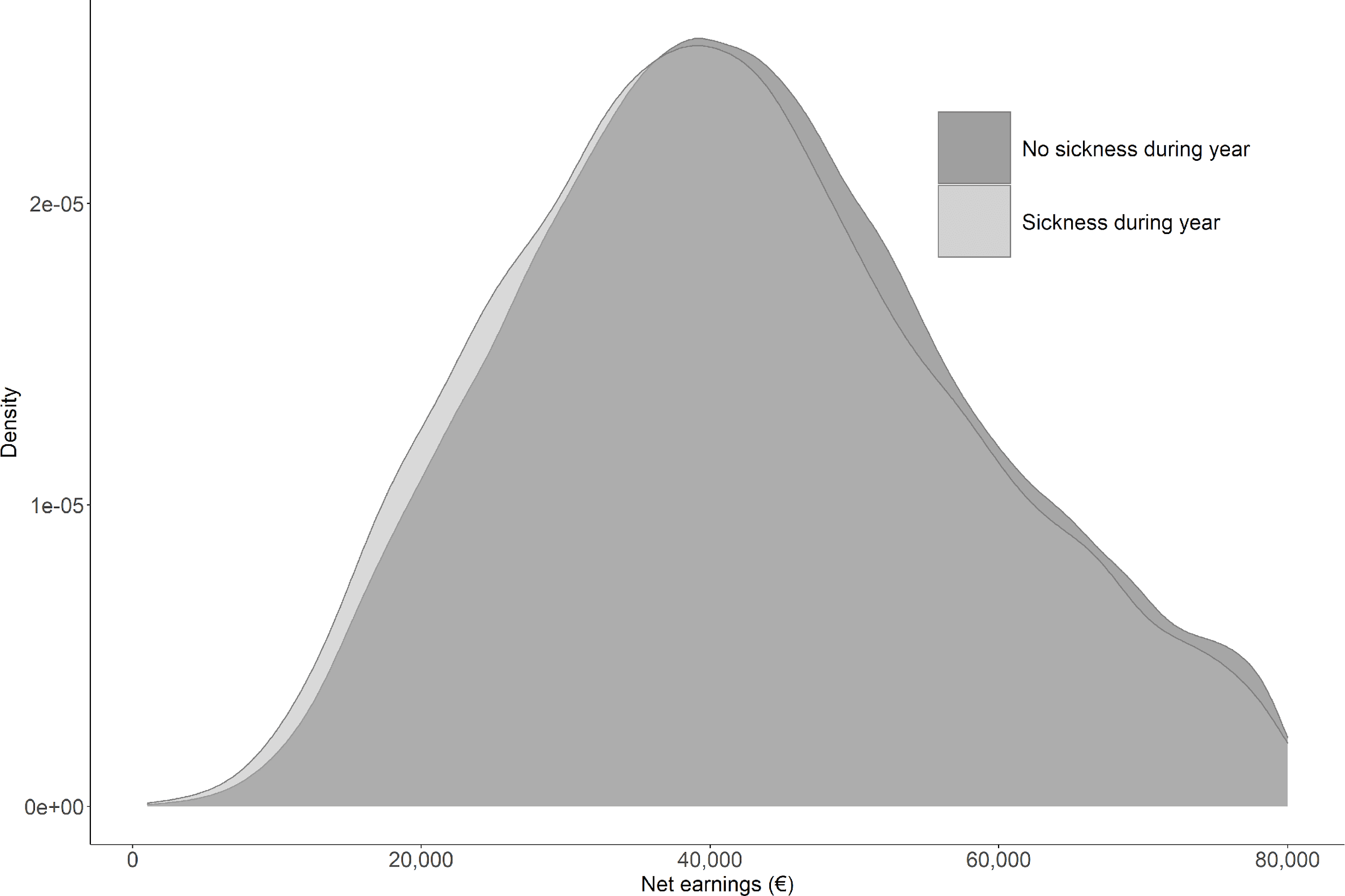
*

*Notes: Sample size: 56,268 year-person observations with no sickness during the year, 10,961 year-person observations with sickness during the year.*

Figure A2. Histogram of life satisfaction by sickness status


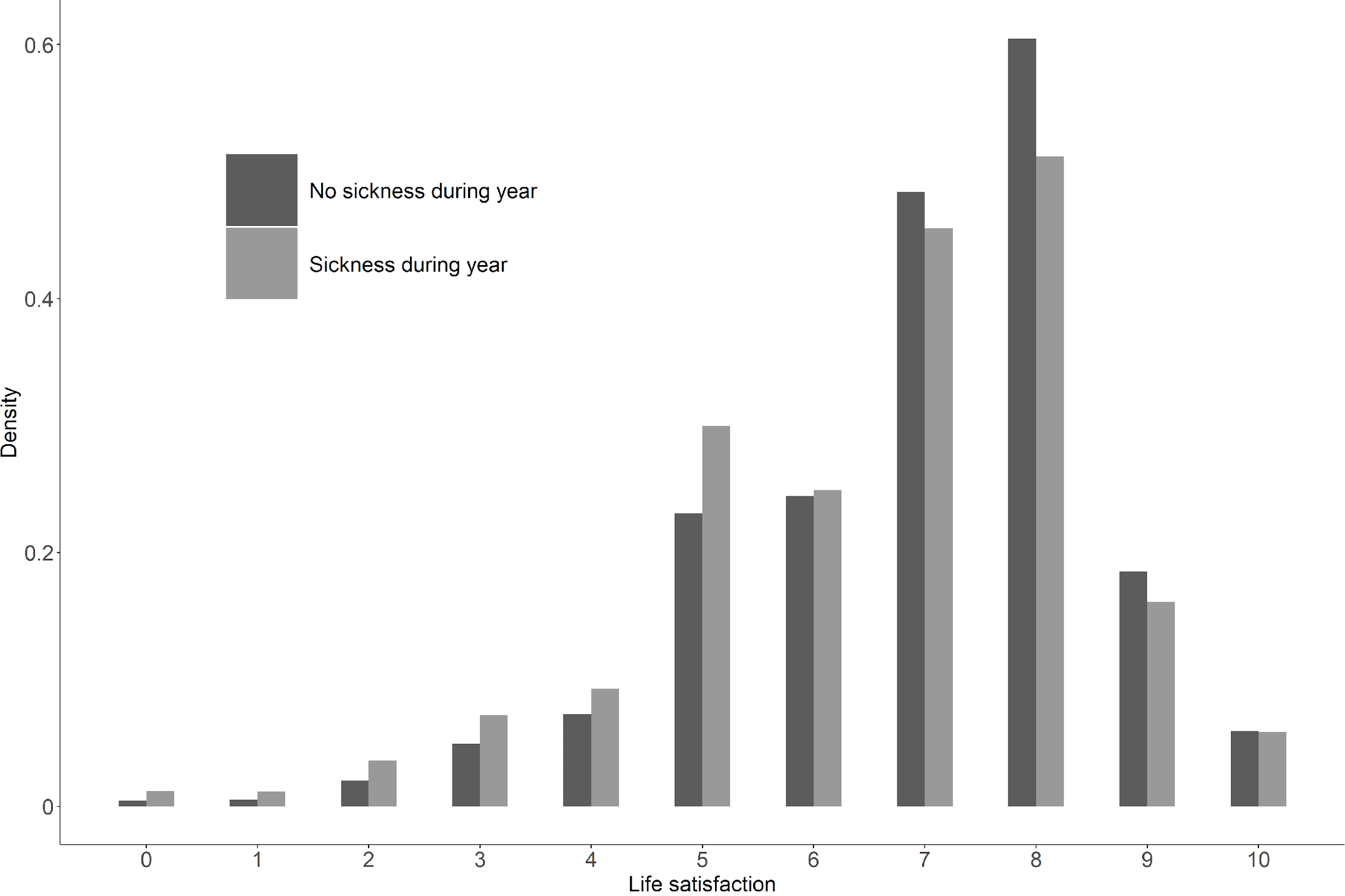


*Notes: Sample size: 56,268 year-person observations with no sickness during the year, 10,961 year-person observations with sickness during the year.*

Figure A3. Estimated relative risk aversion


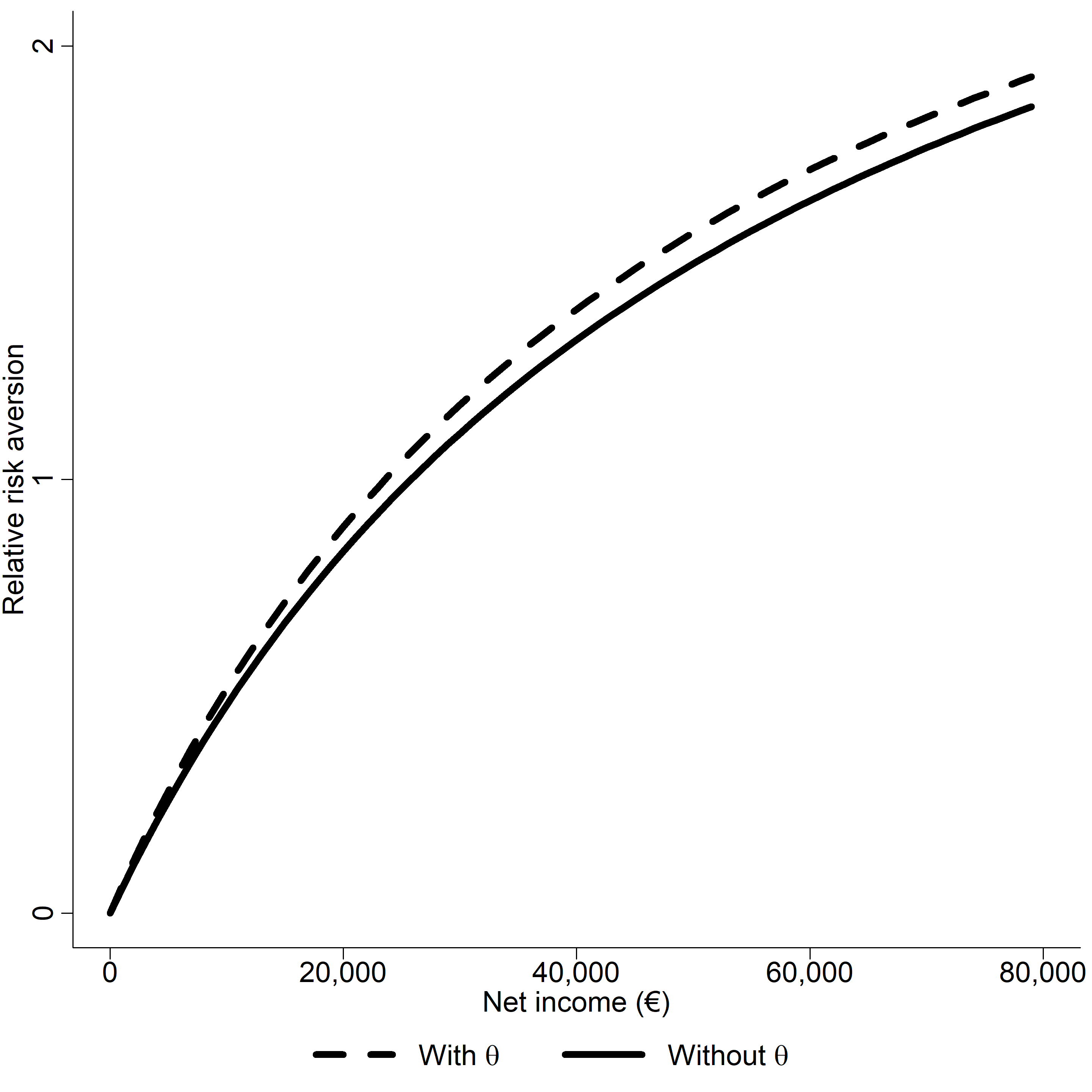


*Notes: The relative risk aversion values are from a HARA utility function with parameter values of* $\left\{ \gamma, \omega, \theta\right\}=\{3.19, -56.49,4.86\}$ *at different levels of disposable equivalized income. which by assumption equals consumption, shown in Table 2, model 2, obtained from estimating equation (6).*

Table A1. Estimates for binarized outcomes

|  | Cutoff at 6+ | Cutoff at 7+ | Cutoff at 8+ | Cutoff at 9+ |
| --- | --- | --- | --- | --- |
|  | (1) | (2) | (3) | (4) |
| Constant ($\alpha$) | 1.37***  (0.04) | 1.39***  (0.06) | 1.21***  (0.08) | 0.61***  (0.13) |
| Scale parameter $(\beta, thousands)$ | 1.15 (6.89) | 0.86 (4.87) | 0.30 (1.89) | 0.036 (0.36) |
| Relative risk aversion parameter $(\gamma)$ | 3.46** (1.69) | 3.23** (1.55) | 2.76* (1.55) | 2.18 (2.02) |
| Institutions parameter $(\omega)$ | -54.61 (42.51) | -57.3 (44.1) | -69.26 (63.36) | -110.15 (168.76) |
| Fixed cost of sickness $(\theta)$ | 2.79** (1.27) | 1.86 (1.40) | -0.75 (2.49) | 0.41 (8.19) |
| Level effect of sickness $(\delta)$ | -0.05*** (0.01) | -0.06*** (0.01) | -0.06*** (0.01) | -0.01 (0.02) |
| Individual controls | Yes | Yes | Yes | Yes |
| Year fixed effects | Yes | Yes | Yes | Yes |
| N | 67,229 | 67,229 | 67,229 | 67,229 |
| Proportion of 1’s in outcome | 0.80 | 0.68 | 0.43 | 0.13 |

*Notes: Statistical significance: * p<0.1; ** p<0.05; *** p<0.01. The non-linear regression is the fit using a modified Levenberg-Marquardt-type algorithm with sampling weights. The standard errors are in parentheses. All models are estimated with equation (6). Individual controls are age, age squared, household size, female dummy, and a dummy for being married. The starting values are*$:\{\alpha=10, \beta=0, \gamma=2.7, \omega=-15, \theta=15, \delta=0\}$ *and zero for all other parameters. For the estimation, the income variable is in thousands of annual euros. The population is limited to “switchers” (i.e., those who had one or more years with sick leave and one or more years without sick leave). To be defined as having sick leave, a person required 6 weeks of absences. The outcome variable is a dummy for having a life satisfaction score of at least 6, 7, 8 or 9, as noted in the column titles.*

Figure A4. Spline and non-linear fit of life satisfaction and net income split by sickness states for binarized outcome variable

*
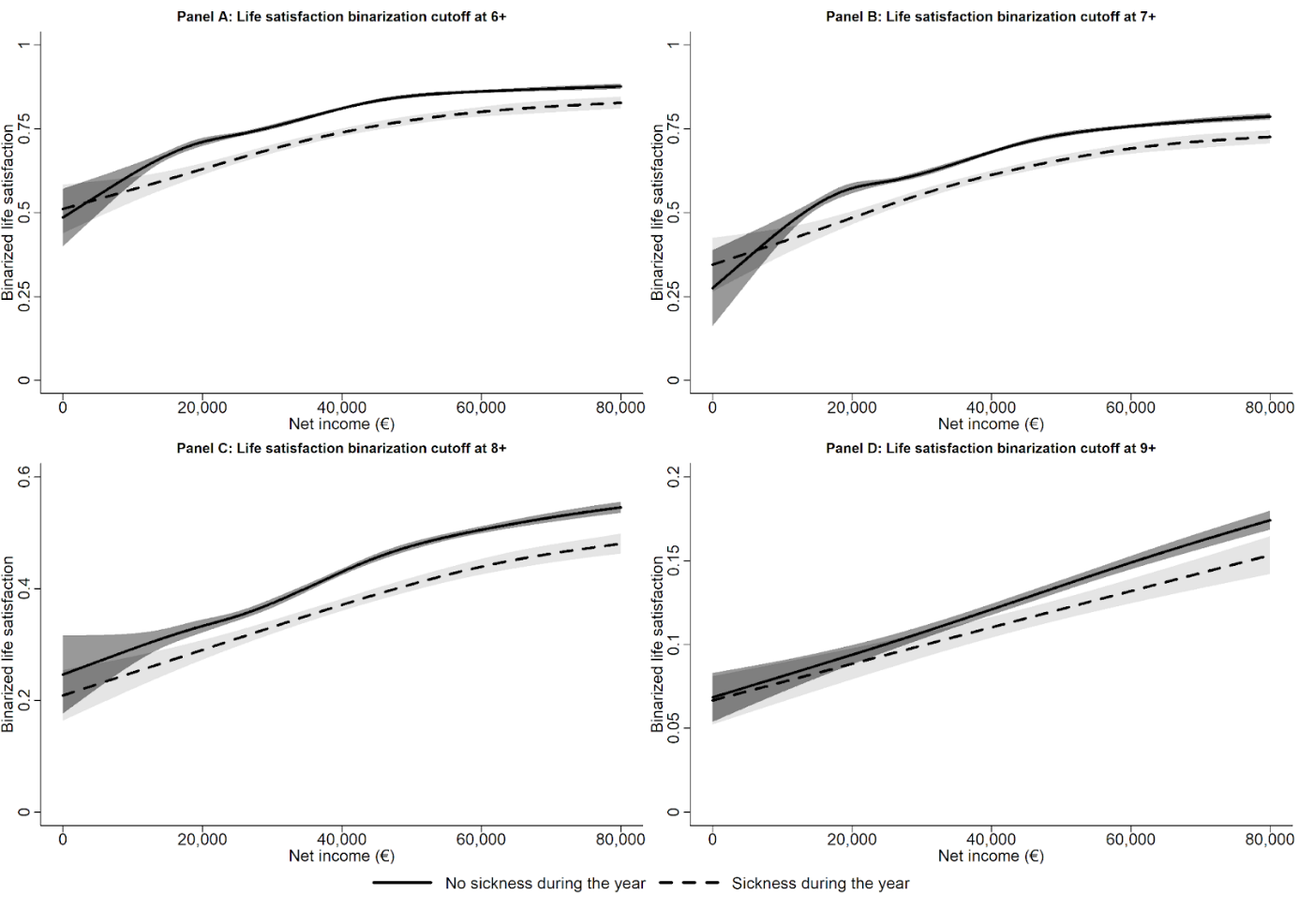
*

*Notes: This figure replicates Figure 3 such that the outcome is binarized at 6 to 9 in panels A to D, respectively. The non-parametric estimate is a spline fit. The x-axis in the figure is truncated at 80,000 euros. The gray area around the curves represents the 95% confidence interval. We omitted the regression fit for clarity. Sample size: 56,268 year-person observations with no sickness during the year, 10,961 year-person observations with sickness during the year.*

Table A2. Estimates for alternative specifications

|  |  | **Changing the sickness variable** | | | | | | |  |  |
| --- | --- | --- | --- | --- | --- | --- | --- | --- | --- | --- |
|  | **Interaction of sickness absence and at least 8 doctor visits** | **At least 8 doctor visits** | **At least 28 doctor visits** | **At least 48 doctor visits** | **Sickness absence of at least 180 days** | **Disability** | **Disability**  **(no controls)** | **Disability**  **(no controls, alternative sample)** | **Main specification (alternative sample)** | **Adaptation** |
|  | (1) | (2) | (3) | (4) | (5) | *(6)* | *(7)* | *(8)* | *(9)* | *(10)* |
| Constant ($\alpha$) | 10.10*** (0.38) | 10.55*** (0.16) | 10.77*** (0.29) | 10.59*** (0.67) | 13.76* (7.70) | 11.10*** (0.78) | 8.29*** (1.01) | 8.28*** (0.99) | 6.54*** (0.24) | 4.40*** (0.18) |
| Scale parameter $(\beta, thousands)$ | 1.31 (10.56) | 0.45 (1.06) | 0.78 (3.53) | 0.16 (0.92) | 0.0013** (0.0005) | 0.68 (6.89) | 0.49 (6.14) | 0.0059 (0.014) | 1.28 (5.90) | 2.75 (35.51) |
| Relative risk aversion parameter $(\gamma)$ | 2.97 (2.19) | 2.58*** (0.60) | 2.70** (1.15) | 2.28 (1.47) | 1.12*** (0.13) | 2.56 (2.42) | 2.43 (2.86) | 1.60** (0.64) | 3.39** (1.52) | 3.41 (3.53) |
| Institutions parameter $(\omega)$ | -51.33 (61.85) | -50.88** (20.89) | -60.44 (40.71) | -47.36 (51.78) | -0.81 (2.25) | -65.78 (104.28) | -76.08 (147) | -6.89 (13.98) | -26.02 (23.39) | -63.1 (101.87) |
| Fixed cost of sickness $(\theta)$ | 3.01 (2.02) | 0.20 (0.78) | 11.08*** (1.75) | 12.87*** (3.36) | 5.96*** (2.14) | -13.19*** (4.46) | -10.69** (5.32) | -3.00 (2.61) | 0.96 (1.08) | 3.21 (2.89) |
| Level effect of sickness $(\delta)$ | -0.29*** (0.06) | -0.20*** (0.02) | 0.05 (0.05) | 0.11  (0.10) | -0.19*  (0.10) | -0.62*** (0.09) | -0.52***  (0.10) | -0.31*** (0.07) | -0.30*** (0.05) | -0.21*** (0.04) |
| Life satisfaction at t-1 |  |  |  |  |  |  |  |  |  | 0.56*** (0.003) |
| Individual controls | Yes | Yes | Yes | Yes | No | Yes | No | No | No | Yes |
| Year fixed effects | Yes | Yes | Yes | Yes | No | Yes | No | No | No | Yes |
| Sample: 50+ years | No | No | No | No | No | No | No | Yes | Yes | No |
| N | 28,096 | 163,570 | 62,524 | 21,539 | 5,448 | 22,785 | 22,785 | 10,203 | 20,100 | *58,905* |

*Notes: Statistical significance: * p<0.1; ** p<0.05; *** p<0.01. The non-linear regression is the fit using a modified Levenberg-Marquardt-type algorithm with sampling weights. The standard errors are in parentheses. All models are estimated with equation (6). Individual controls are age, age squared (except column 9), household size, female dummy, and a dummy for being married. The starting values are*$:\{\alpha=10, \beta=0, \gamma=2.7, \omega=-15, \theta=15, \delta=0\}$ *and zero for all other parameters. For the estimation, the income variable is in thousands of annual euros. The population is limited to “switchers” (i.e., those who had one or more years with and without sickness). The sickness variable by columns is the interaction dummy for being absent from work at least 6 weeks during the year and having at least 8 doctor visits that year (column 1), a dummy for having at least 8, 28, and 48 doctor visits during the year (columns 2, 3 and 4), a dummy for having at least a 180-day sickness absence and disability (columns 6 to 8). In columns 8 and 9, we use an alternative sample of workers above or 50 years of age.*

Figure A5. Spline and non-linear fit of life satisfaction and net income for the disabled and non-disabled states*
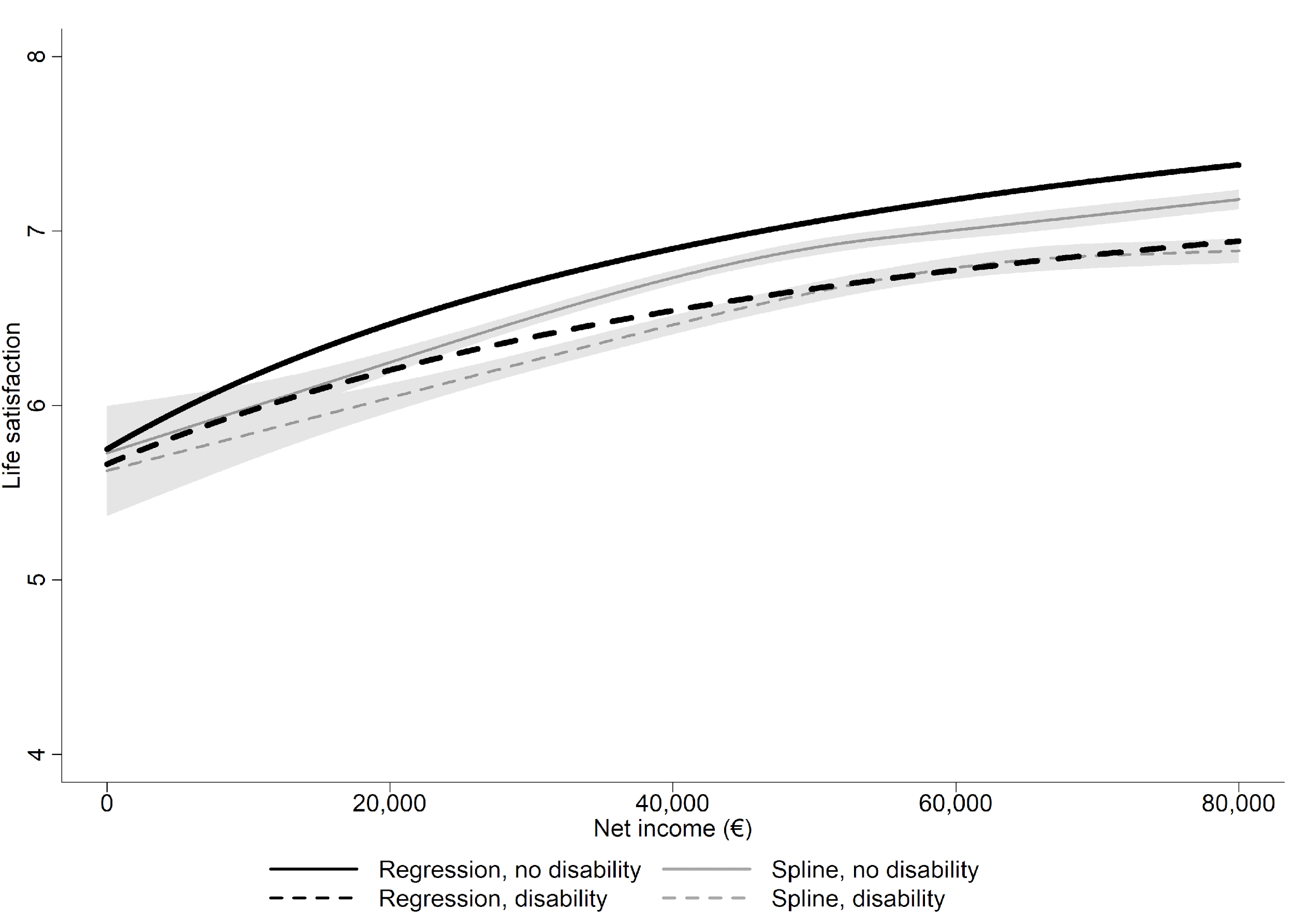
*

*Notes: This figure replicates Figure 3, such that the outcome is disability instead of sickness absence. The non-parametric estimate is a spline fit. The x-axis in the figure is truncated at 80,000 euros. The gray area around the curves represents the 95% confidence interval. The nonlinear regression in the black fit is equation (6), parameter values in Table A1, columns 5 to 8. Sample size: 14,365 year-person observations with no disability, 8,420 year-person observations with disability.* *Parameter values for the regression fit are* $\left\{ \alpha,\beta, \gamma, \omega, \theta,\delta\right\}=\{7.99, 3697.90,2.65,-61.11,-3.07,-0.36\}$*.*

Figure A6. Robustness of estimates to starting values.


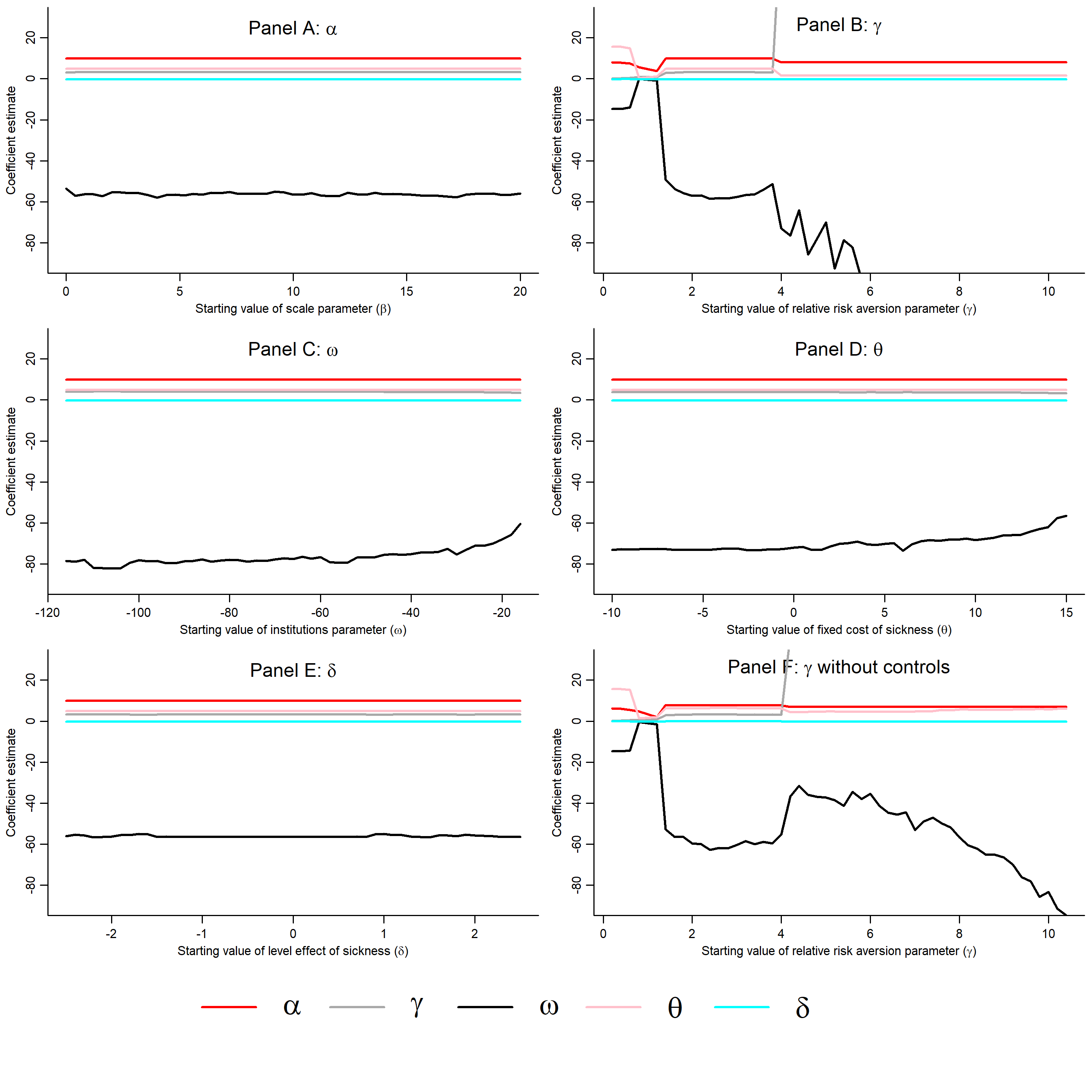


*Notes: The figure shows our main specification (Panels A to E) and main specification without controls (Panel F) with varying starting values. Each panel shows the main parameter values for 51 different starting values. The starting values are spaced equally in Panels A, C, D, and E from 0 to 20 (Panel A), -116 to -16 (Panel C), -10 to 15 (Panel D), and -2.5 to 2.5 (Panel E). In Panels B and F, the starting values vary from 0.2 to 10.4 (skipping the starting value 1). See Table A3, column 4 for the logarithmic specification of γ=1.*

Table A3. Estimates for alternative starting values of the relative risk aversion parameter ($\gamma$)

|  | **Starting** $\boldsymbol{\gamma=2.7}$  **(main specification)** | **Starting** $\boldsymbol{\gamma=5}$ | **Starting** $\boldsymbol{\gamma=0.2}$ | **Logarithmic specification**  **(**$\boldsymbol{\gamma=1}$**)** |
| --- | --- | --- | --- | --- |
|  | (1) | (2) | (3) | (4) |
| Constant ($\alpha$) | 9.93***  (0.20) | 8.11***  (0.11) | 7.96***  (0.46) | 6.49***  (0.20) |
| Scale parameter $\left( \beta\right)$ | 2785.14 (14213.61) | 0 (4.14) | 0.01  (0.00) | 0.69***  (0.04) |
| Relative risk aversion parameter $(\gamma)$ | 3.19**  (1.39) | 148.86 (18034.17) | 0.20***  (0.06) |  |
| Institutions parameter $(\omega)$ | -56.49  (39.35) | -138.3 (16917.33) | -14.71  (36.1) | -3.38**  (1.67) |
| Fixed cost of sickness $(\theta)$ | 4.86***  (1.31) | 1.53  (1.61) | 15.64  (25.45) | 2.41*  (1.35) |
| Level effect of sickness $(\delta)$ | -0.21***  (0.04) | -0.36***  (0.02) | -0.20  (0.25) | -0.28***  (0.04) |
| Individual controls | Yes | Yes | Yes | Yes |
| Year fixed effects | Yes | Yes | Yes | Yes |
| N | 67,229 | 67,229 | 67,229 | 67,229 |
| Residual standard error | 94.55 | 95.66 | 95.15 | 94.63 |

*Notes: Statistical significance: * p<0.1; ** p<0.05; *** p<0.01. The non-linear regression is the fit using a modified Levenberg-Marquardt-type algorithm with sampling weights. The standard errors are in parentheses. All models are estimated using equation (6). Individual controls are age, age squared, household size, female dummy, and a dummy for being married. The starting values for* $\gamma$ *are 2.7, 0.2 and 5 for columns 1 to 3. The starting values for all columns are*$:\{\alpha=10, \beta=0, \gamma=2.7, \omega=-15, \theta=15, \delta=0\}$ *and zero for all other parameters, except for column 4, where* $\gamma$ *is not estimated but assumed at 1. For the estimation, the income variable is in thousands of annual euros. The population is limited to “switchers” (i.e., those who had one or more years with and without sickness).*

**Appendix 1.2: All estimation tables with a loglinear specification**

*In this appendix, we replicate the tables 1, A1 and A2 with a loglinear specification, allowing for the use of individual fixed effects.*

Table A1.2.1. Main estimates with a loglinear specification

|  | **Main specifi-cation** | Years 1992 to 2004 | Years 2005 to 2018 | Years 2015 to 2018 | Main specification without controls |
| --- | --- | --- | --- | --- | --- |
|  | (1) | (2) | (3) | (4) | (6) |
| Log net income | 0.33*** (0.03) | 0.34*** (0.06) | 0.22*** (0.04) | 0.21** (0.10) | 0.60*** (0.01) |
| Sickness | -0.18 (0.12) | 0.15 (0.19) | -0.45*** (0.15) | -0.71*** (0.26) | -0.47*** (0.15) |
| Interaction: Log net income $\times$ sickness | -0.01 (0.03) | -0.08 (0.05) | 0.06 (0.04) | 0.13* (0.07) | 0.05 (0.04) |
| Individual controls | Yes | Yes | Yes | Yes | No |
| Year and individual fixed effects | Yes | Yes | Yes | Yes | No |
| N | 67,229 | 19,395 | 32,423 | 9,302 | 67,229 |

*Notes: This table replicates Table 1 with a loglinear specification. Statistical significance: * p<0.1; ** p<0.05; *** p<0.01. The estimated model is* $LS_{it}=\lambda_{i}+ \gamma_{t}+\beta_{1}Y_{it}+\beta_{2}Sick_{it}+\beta_{3}Y_{it}*Sick_{it}+\boldsymbol{X}_{\boldsymbol{it}}^{\boldsymbol{'}}\delta+\epsilon_{it}$*, where* $LS_{it}$ *is life satisfaction for individual i at time t,* $\lambda_{i}$ *and* $\gamma_{t}$ *are the individual and time fixed effects,* $Y_{it}$ *is log net income and* $\boldsymbol{X}_{\boldsymbol{it}}^{\boldsymbol{'}}$ *is a vector of controls. The standard errors are in parentheses. Individual controls are age, age squared, household size, female dummy, and a dummy for being married. The income variable is in thousands of annual euros. The population is limited to “switchers” (i.e., those who had one or more years with sick leave and one or more years without sick leave). To be defined as having sick leave, a person requires 6 weeks of absences. In columns 2 and 3, we replicate the main specification for years before and after the start of 2005, respectively. In column 4, we exclude years when an individual switches between sickness states. In column 5, we include only those years when an individual switches between sickness states.*

Table A1.2.2. Estimates for binarized outcomes with a loglinear specification

|  | Cutoff at 6+ | Cutoff at 7+ | Cutoff at 8+ | Cutoff at 9+ |
| --- | --- | --- | --- | --- |
|  | (1) | (2) | (3) | (4) |
| Log net income | 0.059*** (0.007) | 0.080*** (0.008) | 0.081*** (0.009) | 0.033*** (0.006) |
| Sickness | -0.057* (0.030) | -0.027 (0.032) | 0.024 (0.032) | 0.021 (0.022) |
| Interaction: Log net income * sickness | 0.004 (0.008) | -0.005 (0.009) | -0.020** (0.009) | -0.010 (0.006) |
| Individual controls | Yes | Yes | Yes | Yes |
| Year and individual fixed effects | Yes | Yes | Yes | Yes |
| N | 67,229 | 67,229 | 67,229 | 67,229 |
| Proportion of 1’s in outcome | 0.80 | 0.68 | 0.43 | 0.13 |

*Notes: This table replicates Table A1 with a loglinear specification. Statistical significance: * p<0.1; ** p<0.05; *** p<0.01. The estimated model is* $LS_{it}=\lambda_{i}+ \gamma_{t}+\beta_{1}Y_{it}+\beta_{2}Sick_{it}+\beta_{3}Y_{it}*Sick_{it}+\boldsymbol{X}_{\boldsymbol{it}}^{\boldsymbol{'}}\delta+\epsilon_{it}$*, where* $LS_{it}$ *is life satisfaction for individual i at time t,* $\lambda_{i}$ *and* $\gamma_{t}$ *are the individual and time fixed effects,* $Y_{it}$ *is log net income and* $\boldsymbol{X}_{\boldsymbol{it}}^{\boldsymbol{'}}$ *is a vector of controls. The standard errors are in parentheses. Individual controls are age, age squared, household size, female dummy, and a dummy for being married. The income variable is in thousands of annual euros. The population is limited to “switchers” (i.e., those who had one or more years with sick leave and one or more years without sick leave). To be defined as having sick leave, a person required 6 weeks of absences. The outcome variable is a dummy for having a life satisfaction score of at least 6, 7, 8 or 9, as noted in the column titles.*

Table A1.2.3. Estimates for alternative specifications with a loglinear specification

|  |  | **Changing the sickness variable** | | | | | | |  |
| --- | --- | --- | --- | --- | --- | --- | --- | --- | --- |
|  | **Interaction of sickness absence and at least 8 doctor visits** | **At least 8 doctor visits** | **At least 28 doctor visits** | **At least 48 doctor visits** | **Sickness absence of at least 180 days** | **Disability** | **Disability**  **(no controls)** | **Disability**  **(no controls, alternative sample)** | **Main specification (alternative sample)** |
|  | (1) | (2) | (3) | (4) | (5) | *(6)* | *(7)* | *(8)* | *(9)* |
| Log net income | 0.371*** (0.045) | 0.302*** (0.018) | 0.305*** (0.031) | 0.306*** (0.054) | 0.45*** (0.121) | 0.3*** (0.056) | 0.67*** (0.031) | 0.704*** (0.046) | 0.269*** (0.056) |
| Sickness | -0.242 (0.195) | 0.074 (0.054) | -0.092 (0.115) | -0.135 (0.217) | -0.845 (0.584) | 0.175 (0.198) | -0.062 (0.187) | 0.065 (0.268) | -0.192 (0.186) |
| Interaction: Log net income * sickness | -0.006 (0.054) | -0.032** (0.015) | 0.010 (0.031) | 0.020 (0.060) | 0.062 (0.162) | -0.111** (0.054) | -0.065 (0.051) | -0.073 (0.072) | -0.012 (0.05) |
| Individual controls | Yes | Yes | Yes | Yes | No | Yes | No | No | No |
| Year and individual fixed effects | Yes | Yes | Yes | Yes | No | Yes | No | No | No |
| Sample: 50+ years | No | No | No | No | No | No | No | Yes | Yes |
| N | 28,096 | 163,570 | 62,524 | 21,539 | 5,448 | 22,785 | 22,785 | 10,203 | 20,100 |

*Notes: This table replicates Table A2 with a loglinear specification. Statistical significance: * p<0.1; ** p<0.05; *** p<0.01. The estimated model is* $LS_{it}=\lambda_{i}+ \gamma_{t}+\beta_{1}Y_{it}+\beta_{2}Sick_{it}+\beta_{3}Y_{it}*Sick_{it}+\boldsymbol{X}_{\boldsymbol{it}}^{\boldsymbol{'}}\delta+\epsilon_{it}$*, where* $LS_{it}$ *is life satisfaction for individual i at time t,* $\lambda_{i}$ *and* $\gamma_{t}$ *are the individual and time fixed effects,* $Y_{it}$ *is log net income and* $\boldsymbol{X}_{\boldsymbol{it}}^{\boldsymbol{'}}$ *is a vector of controls. The standard errors are in parentheses. Individual controls are age, age squared, household size, female dummy, and a dummy for being married. The income variable is in thousands of annual euros. The population is limited to “switchers” (i.e., those who had one or more years with and without sickness). The sickness variable by columns is the interaction dummy for being absent from work at least 6 weeks during the year and having at least 8 doctor visits that year (column 1), a dummy for having at least 8, 28, and 48 doctor visits during the year (columns 2, 3 and 4), a dummy for having at least a 180-day sickness absence and disability (columns 6 to 8). In columns 8 and 9, we use an alternative sample of workers above or 50 years of age.*

Figure A1.2.1. Main model and loglinear fit for the effect of sickness


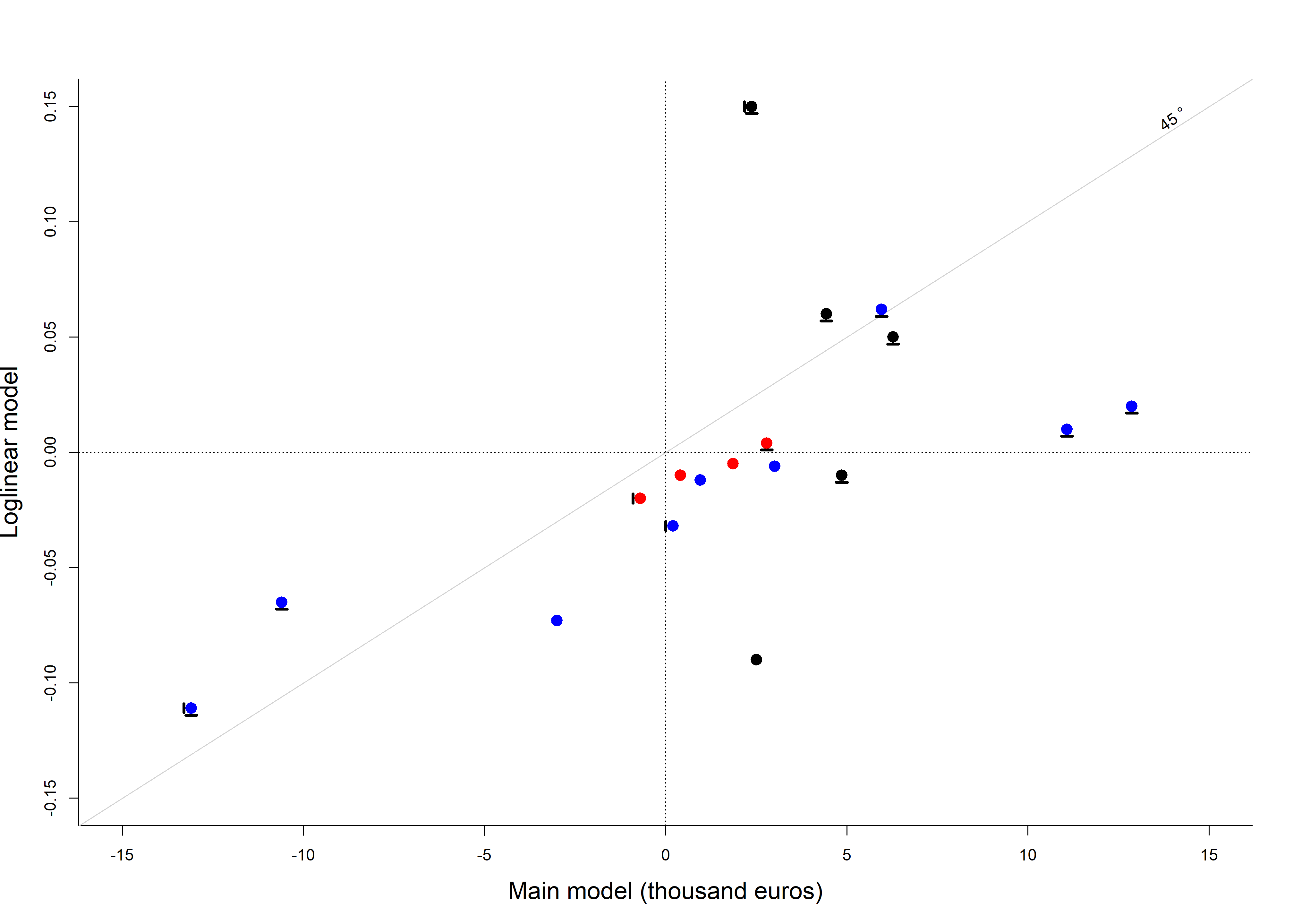


*Notes. Each dot represents the estimated effect of sickness from the main model (Tables 2, A1 and A2, row “Fixed cost of sickness (*$\theta$*)”) and the loglinear model (Tables A1.2.1, A1.2.2 and A1.2.3, row “Interaction: Log net income * sickness”). Black dots are from Tables 2 and A1.2.1, red dots from Tables A1 and A1.2.2. and blue dots from Tables A2 and A1.2.3. The horizontal line below the dot means that the estimate is significant at the 10 % level in the main model. The vertical line on the left side of the dot denotes that the estimate is significant at the 10 % level in the loglinear model.*

Figure A1.2.2. Spline and loglinear fit of life satisfaction and net income split by sickness states


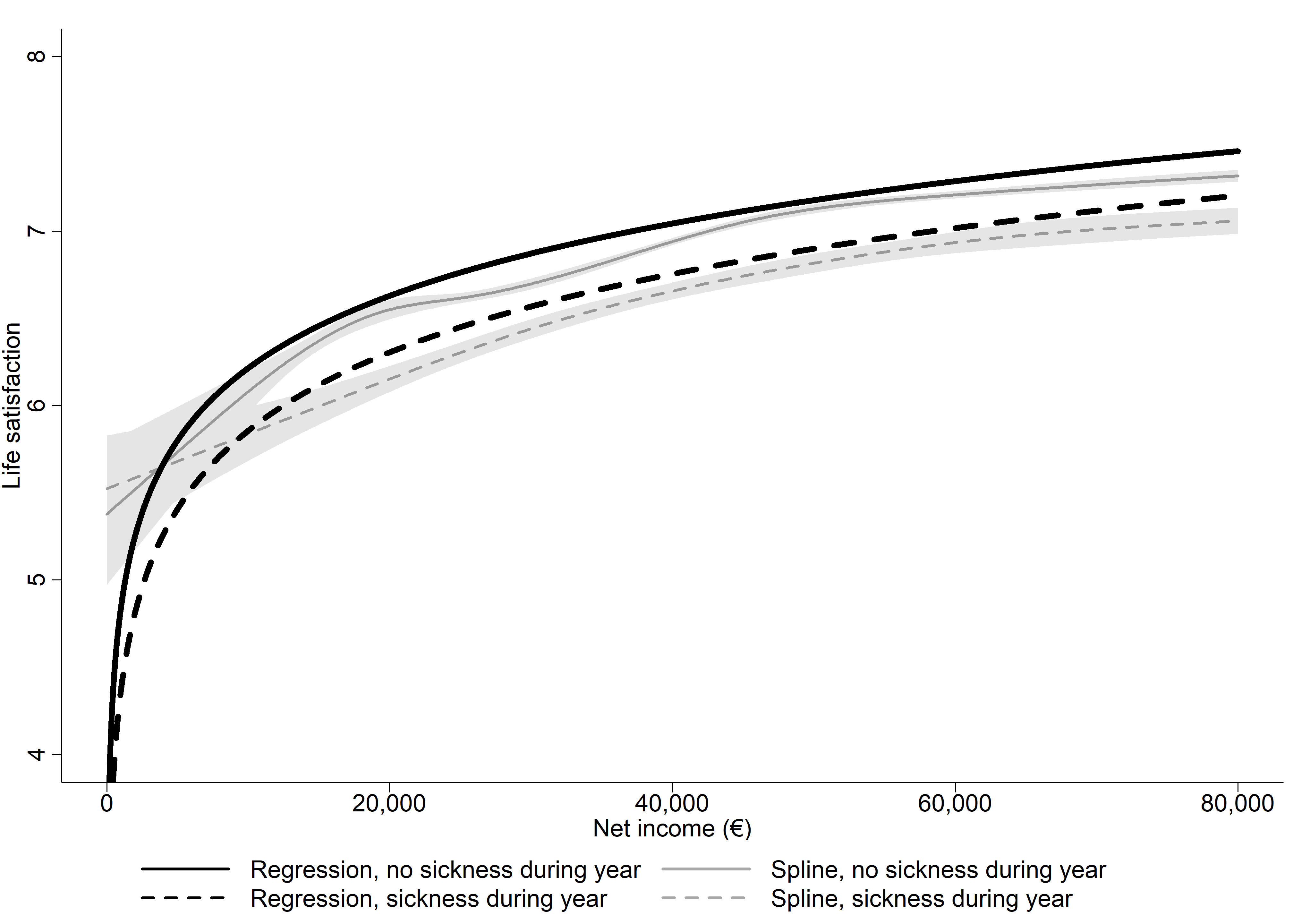


*Notes: The non-parametric estimate is a spline fit. The x-axis in the figure is truncated at 80,000 euros. The gray area around the curves represents the 95% confidence interval. The regression is a loglinear fit of life satisfaction on ne income, sickness and their interaction., parameter values in Table A1.2.1, model 6. Sample size: 56,268 year-person observations with no sickness during the year, 10,961 year-person observations with sickness during the year.*

**Appendix 2: Augmented Baily-Chetty model**

We adapt the canonical Baily-Chetty model of unemployment insurance to sickness insurance (Baily, 1978; Chetty, 2006; Chetty and Finkelstein, 2013). Consider a representative worker who has an initial level of assets $A_{0}$ and wage $w$. Assume that the agent is injured or becomes ill at work with probability $p(E)$, usually denoted $p$. $p(E)$ is a decreasing function of $E$, his chosen sickness-avoidance effort level, with convex effort cost $\psi_{E}(E)$. If the agent is injured or becomes ill, he takes sick leave. In the sick state, there is no risk of repeated sickness or unemployment, and the agent makes no labor supply choices. In the sick state, the agent must be rehabilitated to return to work.

In the sick state, the agent receives a benefit,$b$, for the duration of the sickness benefit and subsequently returns to work. The sickness duration, $D$*,* is assumed to be a choice variable. Non-pecuniary costs and benefits of sickness duration and effort are captured by concave increasing functions $\psi_{D}(D)$ and $\psi_{E}(E)$. Let $k\in\left\{ e,s \right\}$ and $U_{k}(c_{k})$ be strictly concave utility over consumption, where subscripts *e* and *s* stand for being at work and on sick leave, respectively. The utility is assumed to be state-dependent, specifically with a fixed cost of sickness, $u\left( c,1 \right)= u\left( c-\theta\right)+\delta$, $with \delta<0 to allow for u\left( c,1 \right)<u\left( c,0 \right)$. The agent chooses $c_{e}$, $c_{s}$, *E,* and *D* at time 0 to solve,

$$max \left( 1-p(E) \right)u\left( c_{e},0 \right)+p(E)\left( u\left( c_{s},1 \right)+\psi_{D}\left( D \right) \right)-\psi_{E}(E)$$

$$s.t. A_{0}+\left( w-\tau\right)-c_{e}\geq0$$

$$A_{0}+bD+w\left( 1-D \right)-c_{s}\geq0.$$

while taking $\left( b, \tau\right)$ as fixed. This assumption is critical. The social planner chooses the benefits, *b*, that maximize the agent’s indirect utility under the condition that taxes collected $(\tau)$ equal benefits paid. The taxes here are modeled to be lump sum, so they do not affect labor supply choices under no sickness.^^[[1]](#footnote-1)^^ The social planner’s problem, with p*(E),* written as *p*, is as follows:

$$V\left( b,\tau, E \right)$$

$$s.t. \left( 1-p \right)\tau\geq pbD.$$

At the optimum, the optimal benefit rate, *b**, must satisfy the following:

$$\frac{dV(b,\tau, E)}{d(b^{*})}=0,$$

where $\tau$ and E are functions of *b*.

$$V\left( b \right)=\left( 1-p \right)u\left( c_{e},0 \right)+p\left( u\left( c_{s},1 \right)+\psi_{D}\left( D \right) \right)-\psi_{E}\left( E \right)+\lambda_{e}\left[ A_{0}+w-\tau-c_{e} \right]+\lambda_{u}\left[ A_{0}+bD+\left( w-\tau\right)\left( 1-D \right)-c_{s} \right].$$

The function is optimized over ${\{c}_{e}, c_{s}, D, E, \lambda_{e},\lambda_{s}\}$ . We assume that the value function $V\left( b \right)$ is differentiable such that the envelope theorem applies. Thus, following the envelope theorem, changes in the functions have no first-order effect. Specifically, $\frac{dE}{db}=0$, giving the interior optimum as follows:

$\frac{dV(b,\tau, E)}{db(b^{*})}=-\lambda_{e}\frac{d\tau}{db}+\lambda_{s}D=0.$ (A1)

From the agent optimization, we know that:

$\lambda_{e}=\left( 1-p \right)u(c_{e},0)$ and $\lambda_{s}=pu\left( c_{s},1 \right).$ (A2)

From the social planner budget constraint, on which the change in effort does have a first-order effect:

$\frac{d\tau}{db}=\frac{p}{1-p}\left( D+\frac{bdD}{db} \right)+\frac{Db}{\left( 1-p \right)}\frac{dloglog (\frac{p}{1-p})}{db}$ (A3)

Substituting (A3) and (A2) into (A1) yields an implicit equation for the optimal policy (an augmented Baily-Chetty formula):

$\epsilon_{D,b}+\epsilon_{r,b}=\frac{u^{'}(c_{s},1)-u^{'}(c_{e},0)}{{u^{'}(c}_{e},0)}=\frac{u^{'}(c_{s}-\theta,1)-u^{'}(c_{e},0)}{{u^{'}(c}_{e},0)}\approx\gamma\frac{\Delta c+\theta}{c_{e}}\left[ 1+\frac{1}{2}\rho\frac{\Delta c+\theta}{c_{e}} \right]$, (A4)

where$\epsilon_{r,b}=\frac{dloglog (\frac{p}{1-p})}{dloglog (b)}$ is the elasticity of the odds ratio ($r=\frac{p}{1-p}$) of sickness leave with respect to the sickness benefit, i.e., the extensive margin; $\epsilon_{D,b}=\frac{dloglog (D)}{dloglog (b)}$ is the elasticity of the duration of sick leave with respect to the sickness benefit, i.e., the intensive margin; $\frac{\Delta c}{c_{e}}$ is the proportional drop in consumption while on sick leave; $\gamma=-\frac{{C_{e}u}^{''}(C_{e},0)}{u^{'}(C_{e},0)}$ is the coefficient of relative risk aversion; and $\rho=-\frac{{C_{e}u}^{'''}(C_{e},0)}{u^{''}(C_{e},0)}$ is the coefficient of relative prudence. The right-hand side of the formula approximates the increase in relative marginal utility given the drop in consumption under sick leave and yields an implicit equation for the optimal benefit, *b,* which is based on the sufficient statistics approach, $\left( \epsilon_{b}, \frac{\Delta c+\theta}{c_{e}}, \gamma\right), \epsilon_{b}=\epsilon_{D,b}+\epsilon_{r,b}$.

The welfare change can be written in terms of relative marginal utilities of consumption in the two states. If individuals’ behaviors were not distorted by the provision of insurance, the social planner would achieve the first best by setting $b$ to perfectly smooth utilities, ${u^{'}}_{s}\left( c_{s},1 \right)={u^{'}}_{e}(c_{e},0)$. Note that equation (A4) is an implicit one. However, the envelope theorem guarantees that one need not fully characterize all the margins to which individuals can respond to calculate the net welfare gain of social insurance. In particular, all other behavioral responses can be ignored when setting the optimal benefit level, except for the elasticity parameters ($\epsilon_{D,b}$ and$\epsilon_{r,b}$) that enter the government budget constraint directly. However, the social planner cannot directly choose observed consumption levels or $\Delta c$ (hidden savings); rather, it determines the benefit level, which influences income replacement rates, which are observable. Kolsrud et al. (2018) find that the consumption drop increases with the duration of an unemployment spell and that savings and credit play a limited role in smoothing consumption. Equating consumption with income, we can directly solve for the optimal $\frac{\Delta y}{y_{e}}$ using equation (2):

$RR=1-\frac{\Delta y}{y_{e}}=\left( \frac{\omega}{y_{e}}+\frac{\theta}{y_{e}} \right)+{\left( 1-\frac{\omega}{y_{e}} \right)\left( 1+{\epsilon_{r,b}+\epsilon}_{D,b} \right)}^{-\frac{1}{\gamma}},$ (A5)

We employ the form $u\left( y,S \right)= u\left( c\left( y \right)-\theta S \right)$ as a simple parametrization of the state-dependent utility of the qualitative type we have observed in Figure 1. The social planner now must consider $\theta$ in addition to the standard Baily-Chetty parameters {$\epsilon_{b}, \gamma, \rho$} for optimal policy. The relationship observed by Finkelstein et al. (2013) would require an alternative functional form.

The envelope theorem plays a critical role in generalizing (A4) with minor modifications to more realistic dynamic models with endogenous savings and borrowing constraints (Chetty and Finkelstein, 2013). One could also complement the model following Kolsrud et al. (2018), who model the effect of duration-dependent benefit rates in unemployment.

In the standard Baily-Chetty formula, a non-linear benefit rule could possibly be optimal if risk aversion or the incentive effect varies significantly according to the income level. Additionally, if the aim of the insurance scheme is to contribute to the redistribution of income from rich to poor households, a non-linear benefit rule might be well motivated.

**Appendix 3: Sickness insurance in Europe**

MISSOC (2017) comparative tables describe the European sickness insurance schemes (cf. Frick and Malo, 2008). The tables distinguish at least five dimensions, in which the schemes differ. Two of the key dimensions are depicted in Figure A3.1. The crucial aspect in any social insurance system is the replacement rate (i.e., the rate at which pre-sickness income is covered by sickness insurance). The replacement rates in Europe vary from 50% (Italy, Greece, France, and Austria) to 100% (Luxembourg and Norway). However, some European countries (Iceland, Ireland, Malta, and the UK) have a lump-sum benefit. Lump-sum benefits imply highly regressive replacement rates and are therefore not shown in Figure A3.1.

The other important dimension presented in Figure A3.1 is the waiting period. A waiting period is the amount of time the person must pass on sick leave before being eligible for the benefit. The waiting periods vary between 0 and 3 days in the countries with proportional replacement rates. Three-day waiting periods are found in Southern Europe, the Czech Republic, and Estonia. Northern European countries tend to have no waiting periods at all. The waiting period plays a large role in short sickness spells.

The other three dimensions in which European sickness insurance schemes differ are coverage, maximum duration, and qualifying period. Coverage is broad for full-time employees in all countries in Europe and varies primarily in terms of how the self-employed are treated. Maximum durations vary slightly between countries such that the mode is at one year. The qualifying periods (i.e., the time required at the job before eligibility) vary from none to 6 months.

To capture within-country heterogeneity in the replacement rates, Figure A3.1 is insufficient. Some countries, such as Finland, have notably non-linear benefit rules. The benefit curves for Germany, France, and Finland are depicted in Figure A3.2.

Figure A3.1. Characteristics of sickness insurance schemes in Europe


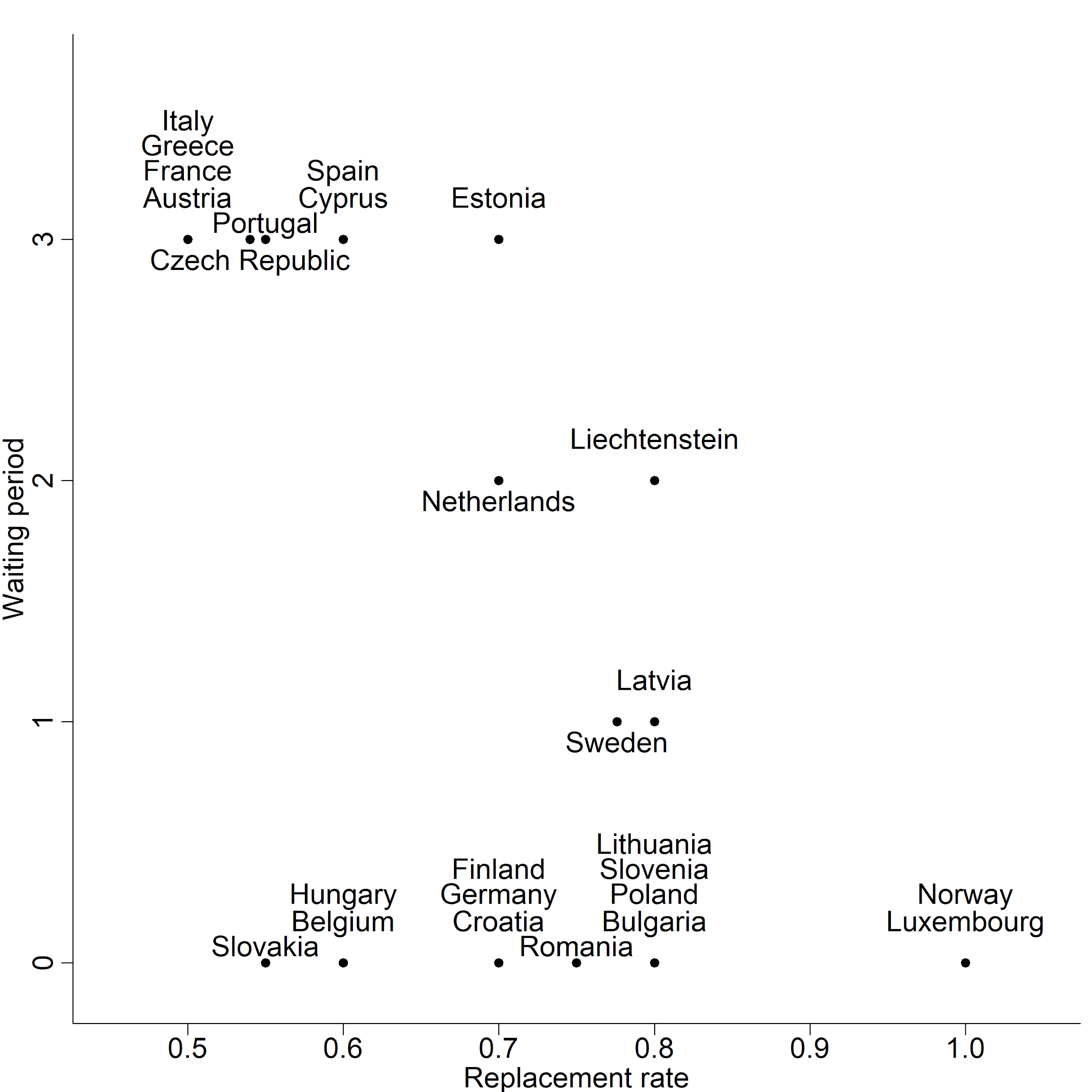


*Notes: Single, lowest income bracket, initial benefit level, and the general case for long-term employment. Denmark: not defined in the table. Iceland, Ireland, Malta and the UK: lump sum benefit. Switzerland: varies by individual contract. Source: MISSOC comparative table, 2017/07/01.*

Figure A3.2. Generosity of sickness insurance schemes in three European countries


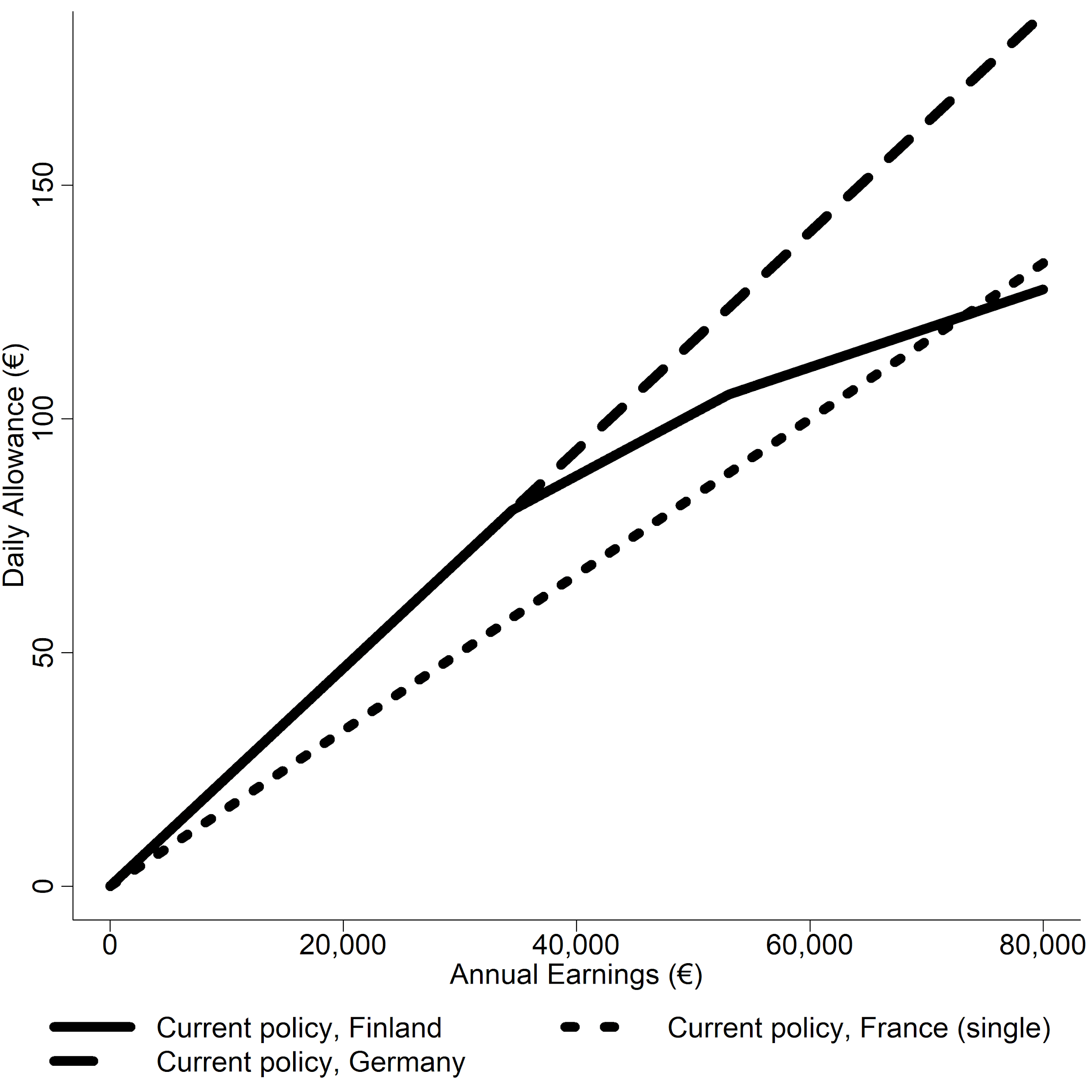


**Appendix 4. Replication of main results using EU-SILC cross-sectional data**

We replicate the main results using the data from the EU Statistics on Income and Living Conditions (EU-SILC). The EU-SILC is a harmonized dataset on income, social inclusion, and living conditions that covers the material and subjective aspects of well-being.^^[[2]](#footnote-2)^^ The EU-SILC data are based on a combination of survey and register-based information, depending upon the source country. We use the 2013 data for all 27 countries that were members of the European Union in 2013 (see Appendix 3 for a description of sickness insurance institutions in Europe). In addition, we use data on Iceland, Norway, and Switzerland, for a total of 30 countries.

Descriptive statistics are presented in Table A4.1. The subsample that is employed is large (~125,000), whereas the subsample for those on sick leave is substantially smaller (~1,200). Life satisfaction runs from 0 to 10. Mean life satisfaction is ~0.8 points lower for those on sick leave.

For the EU-SILC, we use four variables to construct our estimates. To define the working population, we restrict the employed sample to those aged 18 to 64 years who usually work more than 30 hours per week (the variable PL060 in EU-SILC). We define the sick leave population as those who usually work less than 30 hours per week due to “disability or illness” (PL120). Our measure of sickness absence thus captures more longer-term sickness that causes the largest financial burden to the health care system. Note that our variable does not allow us to separate the effect of sickness from that of sickness absence. We thus use both terms interchangeably. Our focus is on the policy, which is conditional on sickness absence. The measure of subjective well-being is life satisfaction. It is the best available survey measure of decision utility (Benjamin et al., 2012; 2014a-2014b; for discussion of decision versus experienced utility, see Kahneman et al., 2007). We use the standard life satisfaction question (PW010): “Overall, how satisfied are you with your life nowadays?” For income, we use the PPP-adjusted equivalized disposable household income per consumption unit (HX090; see Section 3.4 for a discussion of consumption vs. income).

Using EU-SILC data, Tables A4.1 and A4.2 and Figures A4.1 to A4.4. replicate the main Tables 1 and 2 and Figures 1 to 4 done with the SOEP panel data and discussed in the main text. The results qualitatively concur with the panel estimate with SOEP data. However, since the population is all of Europe and the sickness state parameters are estimated with a different population in the cross-section, we observe that the fixed cost of sickness at around 11 thousand euros is higher than the main estimate we obtain with SOEP data. The difference could partly stem from the fact that, in the EU-SILC cross-section, the sick individuals are not the same as the those in the healthy state, unlike in the SOEP main estimations. We repeat the analysis at the country level and study the correlation of the institutions parameter estimates with those of institutional variables in Appendix 5.

**Figures and Tables**

Table A4.1. Descriptive statistics

|  | Employed | | On sick leave | |
| --- | --- | --- | --- | --- |
|  | Mean | SD | Mean | SD |
| Life satisfaction | 7.38 | 1.79 | 6.56 | 2.20 |
| Equivalized disposable income (thousands €) | 19.94 | 15.07 | 18.39 | 11.59 |
| Age | 43.77 | 10.77 | 51.18 | 8.95 |
| Female | 0.45 | 0.50 | 0.70 | 0.46 |
| Tertiary education | 0.69 | 0.46 | 0.42 | 0.51 |
| N | 125,166 | - | 1,236 | - |

*Notes: All variable means show statistically significant differences between the groups at the 5% level.*

Table A4.2. Estimation.

| Variable | Estimate |  |
| --- | --- | --- |
| Scale parameter $(\beta)$ | 2.85***  (2.10) | |
| Relative risk aversion parameter $(\gamma)$ | 1.49***  (0.04) | |
| Institutions parameter $(\omega)$ | -22.37***  (3.18) | |
| Fixed cost of sickness $(\theta)$ | 11.37***  (1.93) | |
| Level effect of sickness $(\delta)$ | -0.28**  (0.12) | |
| N | 126,402 | |

*Notes: Statistical significance: * p<0.1; ** p<0.05; *** p<0.01. The non-linear regression is the fit using a modified Levenberg-Marquardt-type algorithm with sampling weights. The standard errors are in parentheses. All models are estimated with equation (6) with* $\alpha$ *set at 10. The starting values, where applicable, are*$:\{\beta=0, \gamma=1.4, \omega=-15, \theta=15, \delta=0\}$*. For the estimation, the income variable is in thousands of annual euros.*

Figure A4.1. Spline fit of life satisfaction and income in Europe and UK, employed vs. sick


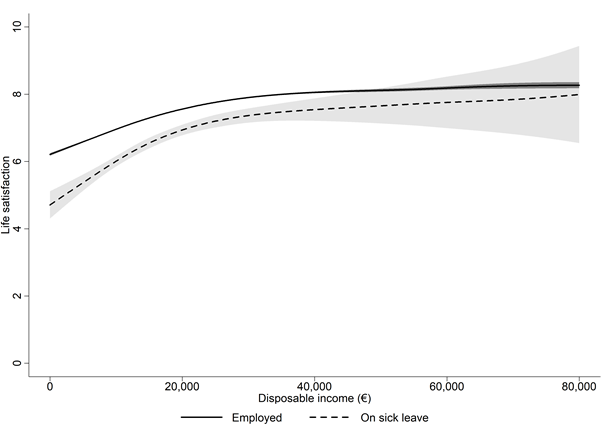


*Notes: The estimates are cubic splines with six knots estimated using the R package “bigsplines” for a cross-section and the following parameter values: unrounded, no manual tuning, unique knots assuming Gaussian standard errors. The gray area around the curves represents the 95% confidence interval. Sample size: 125,166 in employment, 1,236 on sick leave.*

Figure A4.2. Spline and non-linear fit of life satisfaction and income in Europe, employed vs. sick


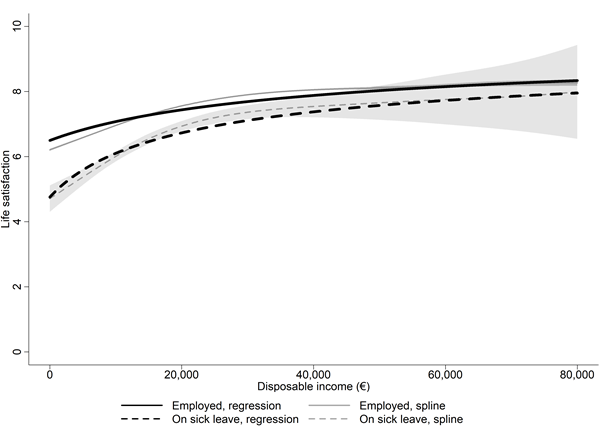


*Notes: The non-parametric estimate is a spline fit. The x-axis in the figure is truncated at 80,000 euros. The non-linear regression fit is equation (6) with* $\alpha$ *set at 10, parameter values in Table 2, model 1, estimated using the whole income distribution. Sample size: in employment 125,166, on sick leave 1,236.*

Figure A4.3. Optimal replacement rates


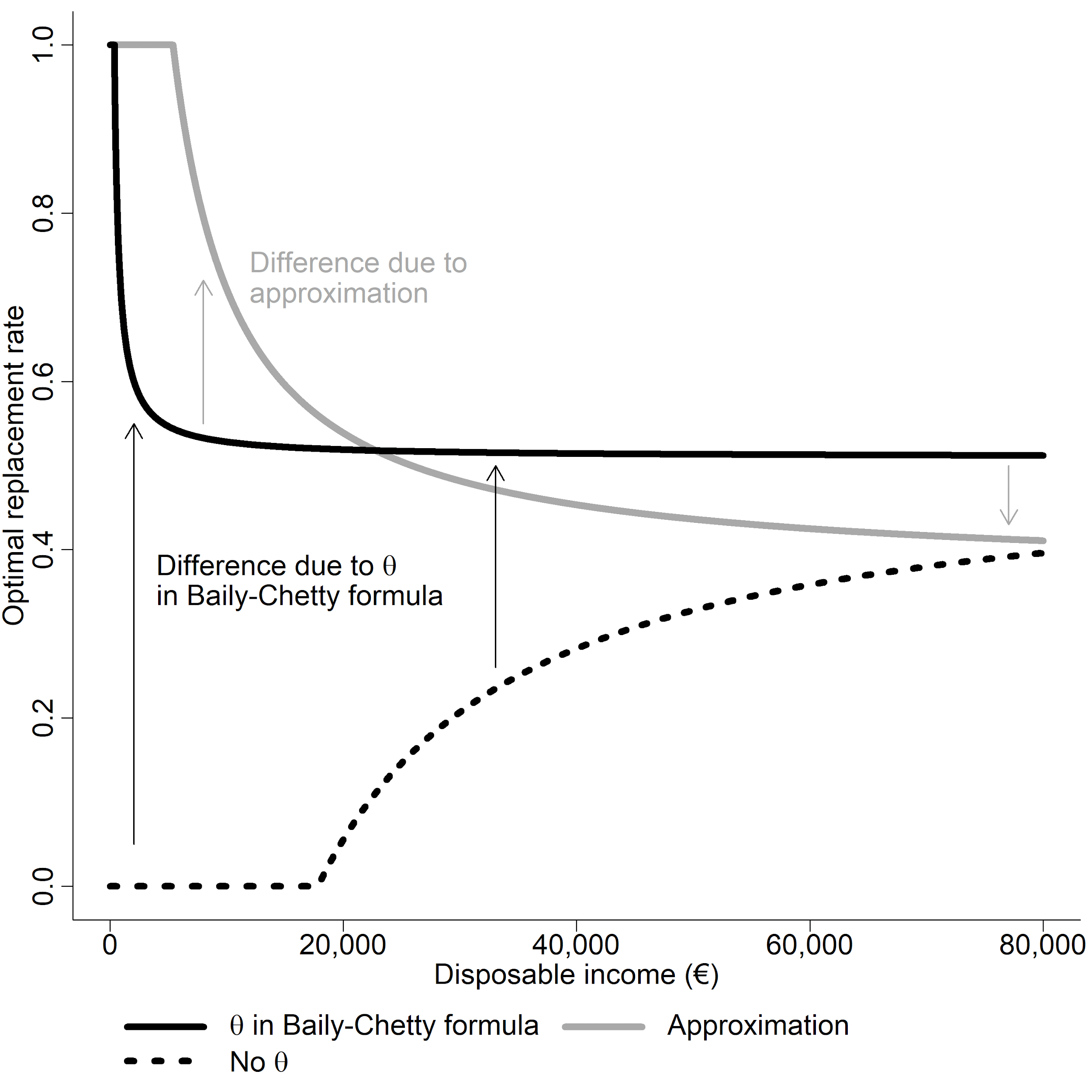


*Notes: The optimal replacement rates are calculated using equation 5. The relative risk aversion values are from a HARA utility function with the parameter values of* $\left\{ \gamma, \omega, \theta\right\}=\{1.36, -18.5, 9.3\}$ *at different levels of disposable equivalized income, which by assumption equals consumption, as shown in Table A4.2, obtained from estimating equation (6) with European data.* $\theta$ *is the fixed cost of sickness, which affects the optimal replacement rate through relative risk aversion (RRA) and the augmented Baily-Chetty formula. Additionally, we assume that* ${\epsilon_{r,b}+\epsilon}_{D,b}=1.5$*.*

Figure A4.4. Prevailing universal sickness insurance policy and estimated optimal curves

*
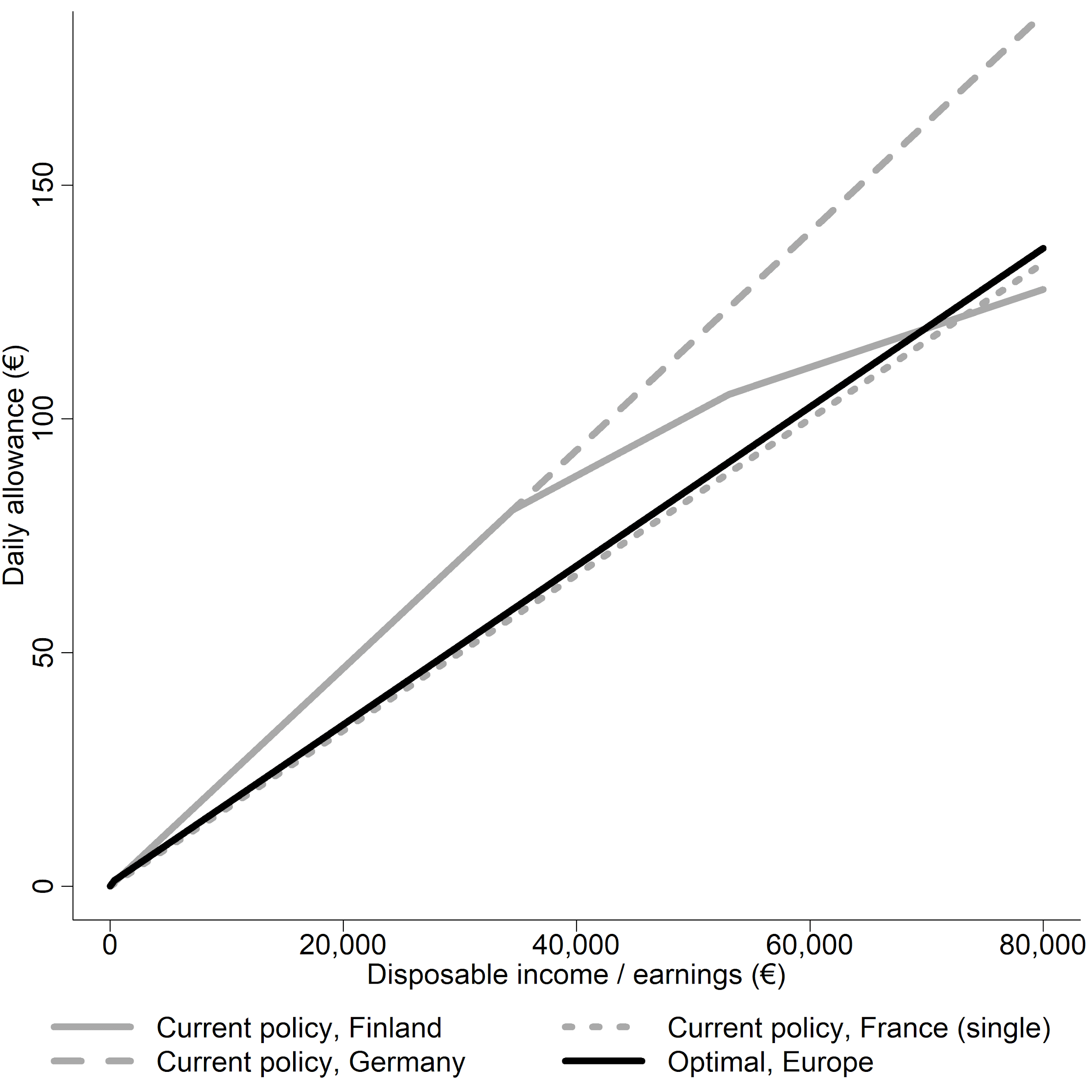
*

*Notes: The optimal replacement rates are calculated using equation 5. The relative risk aversion values are derived from a HARA utility function with the parameter values of* $\left\{ \gamma, \omega, \theta\right\}=\{1.36, -18.5, 9.3\}$ *at different levels of disposable equivalized income, which by assumption equals consumption, as shown in Table A4.2, obtained from estimating equation (6) with European data.* $\theta$ *is the fixed cost of sickness, which affects the optimal replacement rate through relative risk aversion (RRA) and the augmented Baily-Chetty formula. Additionally, we assume that* ${\epsilon_{r,b}+\epsilon}_{D,b}=1.5$*. “Single” refers to a one-member household. The optimal curve is based on equivalized disposable income, whereas the current policy curves are based on earnings.*

**Appendix 5. Country-specific estimates of** $\omega$ **(i.e., the value of institutions) with EU-SILC cross-sectional data**

We replicate the European analysis shown in Appendix 4, now at the country level. Figure A5.1 presents country-level profiles for the relationship between equivalized disposable household income per consumption unit and life satisfaction as a spline fit using the EU-SILC cross-sectional data. The figures also show the fit of equation (6), where only $\omega$ and $\theta$ are allowed to vary and all other parameters are held constant at the values presented in Table A4.2. Table A5.1. shows the sample size by each country.

The functional fit follows the pattern of the aggregate fit remarkably well, given the smaller sample size for each country. The estimation of $\omega$ is robust to the exclusion of low-income individuals in the data because the whole range of incomes is used for the estimation. However, the non-parametric spline is inaccurate for most countries due to the small sample size.

We extract the $\omega$ parameter point estimates from the country-level fits and correlate them with the measures of institutions (Figure A5.2). The $\omega$ parameter is equivalent to giving each citizen an equal increase in income, increasing utility at all income levels. The $\omega$ parameter, which measures this shared increase in utility, captures the value of *all* the characteristics of a country, including, for example, its institutions, social norms, culture, and geographical features. For brevity, we call $\omega$ the institutions parameter. In contrast to the parameter used by Jones and Klenow (2016), the $\omega$ parameter abstracts from consumption levels.

We find that the Nordic welfare states have a high $\omega$. By contrast, high-income Southern European countries have a low $\omega$. The high correlation coefficient between the institutions parameter and trust is notable, at 0.80 for interpersonal trust and 0.82 for the mean trust in the police, the legal system, and the political system. Additionally, the Gini coefficient of equivalized disposable income has a highly significant correlation with the institutions parameter, at -0.59. This high correlations suggest that the institutions parameter captures something that is of real-world significance. However, one should interpret numeric values with caution, even when the rankings and relative values appear to matter.

The sickness benefit can affect the value of institutions and the functional form between income and life satisfaction across countries. However, the correlation coefficient between the replacement rate and the estimated value of institutions is low at 0.20 and not statistically significant. Table A5.2. reports the estimated contribution of institutions and equivalized income to the mean utility by country.

Figure A5.1. Spline and non-linear regression fit of life satisfaction and income by country, employed vs. sick

**
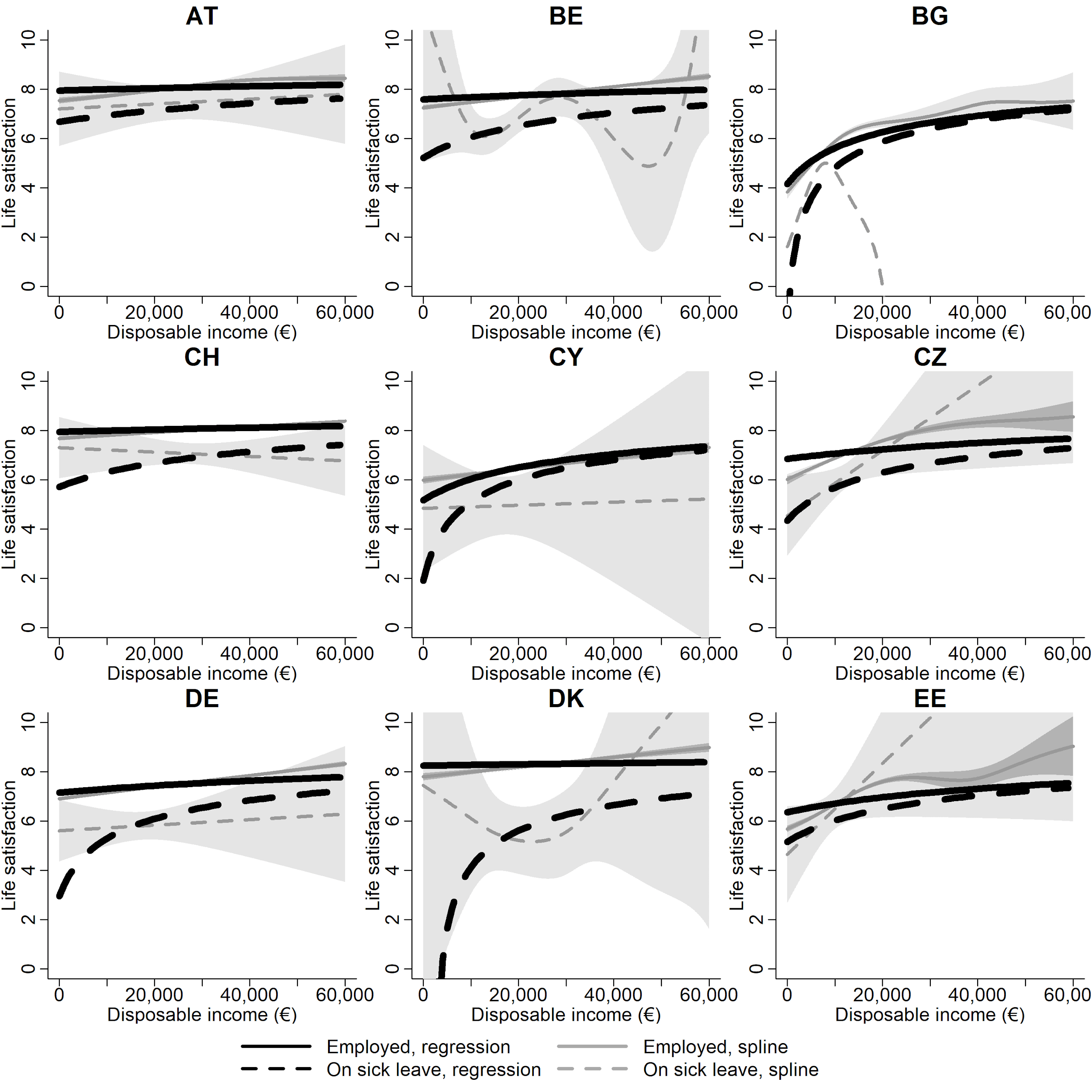
**

*Notes: The estimate is a spline fit. The fit is performed using the whole income distribution, although the x-axis in the figure is truncated at 60,000 euros. Country codes: AT=Austria, BE=Belgium, BG=Bulgaria, CH=Switzerland, CY=Cyprus, CZ=Czech Republic, DE=Germany, DK=Denmark, EE=Estonia.*

Figure A5.1 (cont.). Spline and non-linear regression fit of life satisfaction and income by country, employed vs. sick

**
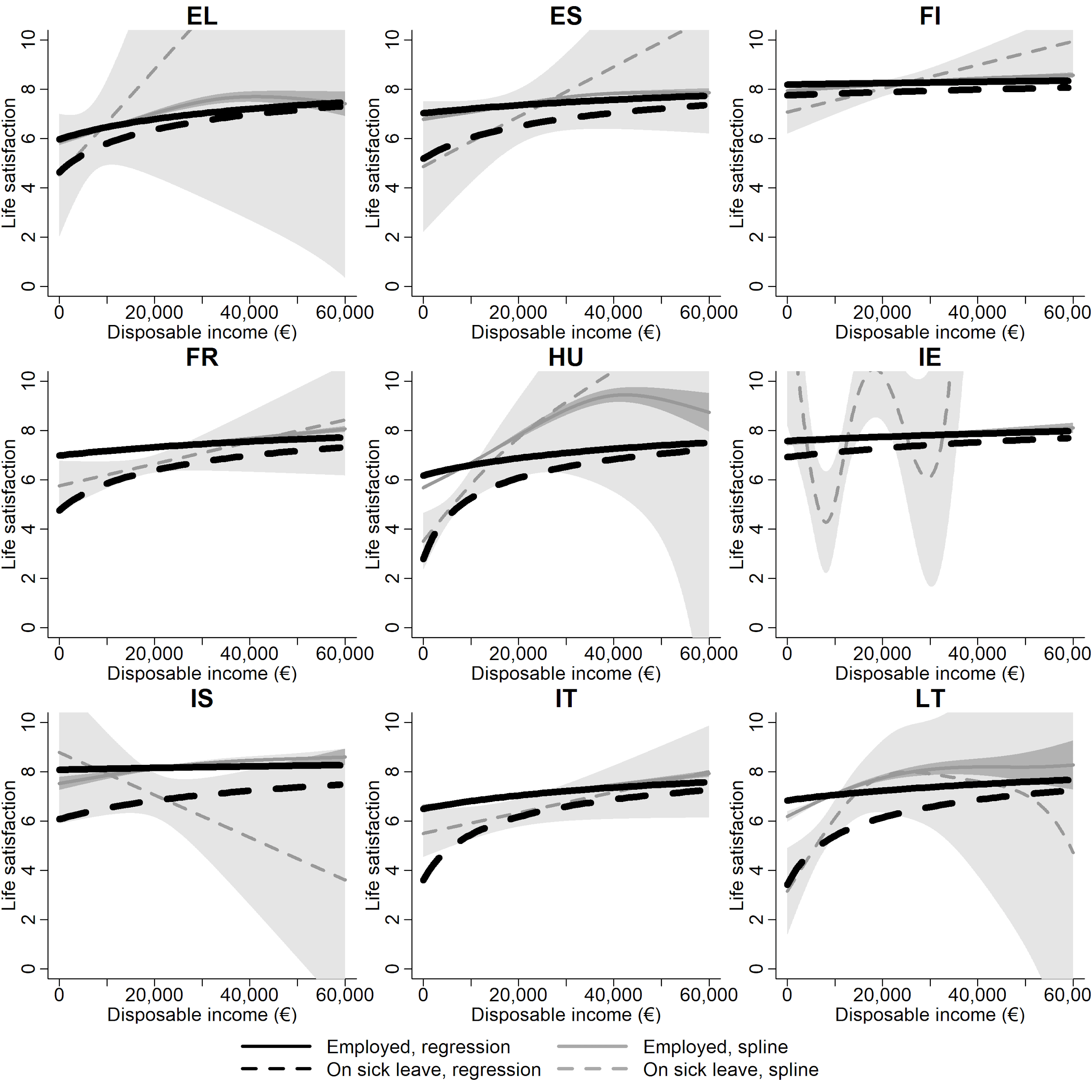
**

*Notes: The estimate is a spline fit. The fit is performed using the whole income distribution, although the x-axis in the figure is truncated at 60,000 euros. Country codes: EL=Greece, ES=Spain, FI=Finland, FR=France, HU=Hungary, IE=Ireland, IS=Iceland, IT=Italy, LT=Lithuania.*

Figure A5.1 (cont.). Spline and non-linear regression fit of life satisfaction and income by country, employed vs. sick

*
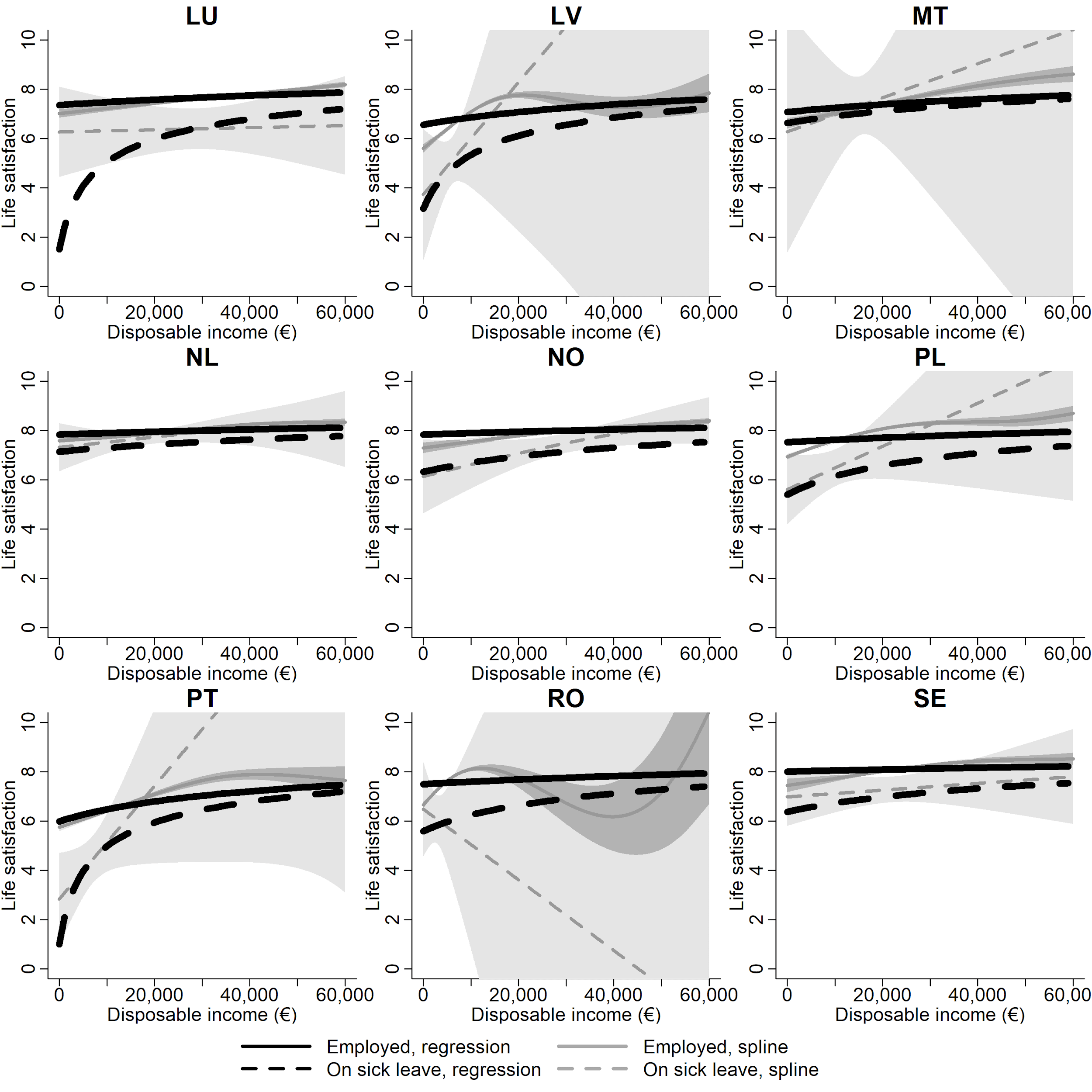
*

*Notes: The estimate is a spline fit. The fit is performed using the whole income distribution, although the x-axis in the figure is truncated at 60,000 euros. Country codes: LU=Luxembourg, LV=Latvia, MT=Malta, NL=Netherlands, NO=Norway, PL=Poland, PT=Portugal, RO=Romania, SE=Sweden.*

Figure A5.1 (cont.). Spline and non-linear regression fit of life satisfaction and income by country, employed vs. sick

*
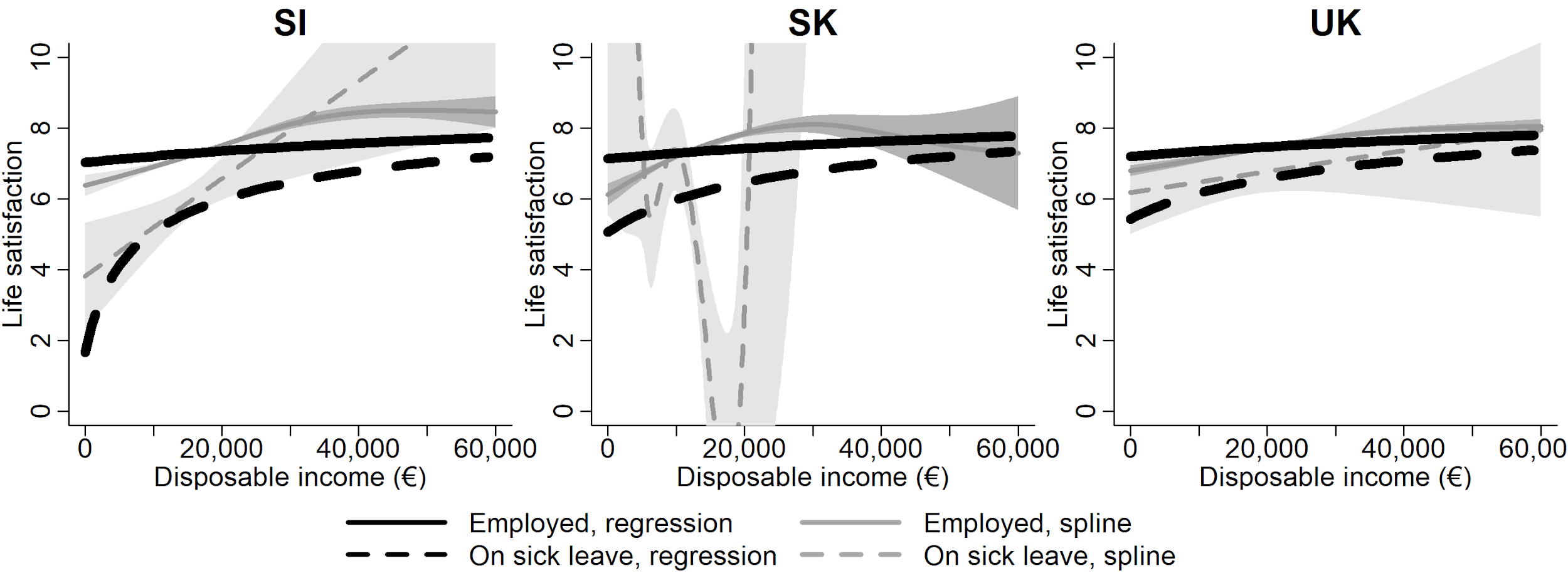
*

*Notes: The estimate is a spline fit. The fit is performed using the whole income distribution, although the x-axis in the figure is truncated at 60,000 euros. Country codes: SI=Slovenia, SK=Slovakia, UK=United Kingdom.*

Table A5.1. Sample size by country and subset

|  | Employed | On sick leave |
| --- | --- | --- |
| Austria | 3,805 | 36 |
| Belgium | 3,521 | 39 |
| Bulgaria | 2,757 | 3 |
| Switzerland | 5,143 | 95 |
| Cyprus | 3,944 | 18 |
| Czech Republic | 4,357 | 45 |
| Germany | 6,857 | 55 |
| Denmark | 2,428 | 14 |
| Estonia | 4,077 | 33 |
| Greece | 3,782 | 16 |
| Spain | 7,757 | 17 |
| Finland | 4,782 | 40 |
| France | 5,605 | 89 |
| Hungary | 6,416 | 94 |
| Ireland | 1,637 | 11 |
| Iceland | 1,429 | 15 |
| Italy | 7,984 | 39 |
| Lithuania | 2,839 | 38 |
| Luxembourg | 1,946 | 31 |
| Latvia | 3,257 | 16 |
| Malta | 2,179 | 6 |
| The Netherlands | 3,740 | 99 |
| Norway | 3,046 | 98 |
| Poland | 7,609 | 31 |
| Portugal | 3,649 | 21 |
| Romania | 4,767 | 14 |
| Sweden | 2,606 | 55 |
| Slovenia | 3,722 | 81 |
| Slovakia | 4,729 | 16 |
| United Kingdom | 4,796 | 71 |

Figure A5.2. Country-level scatter plots

**
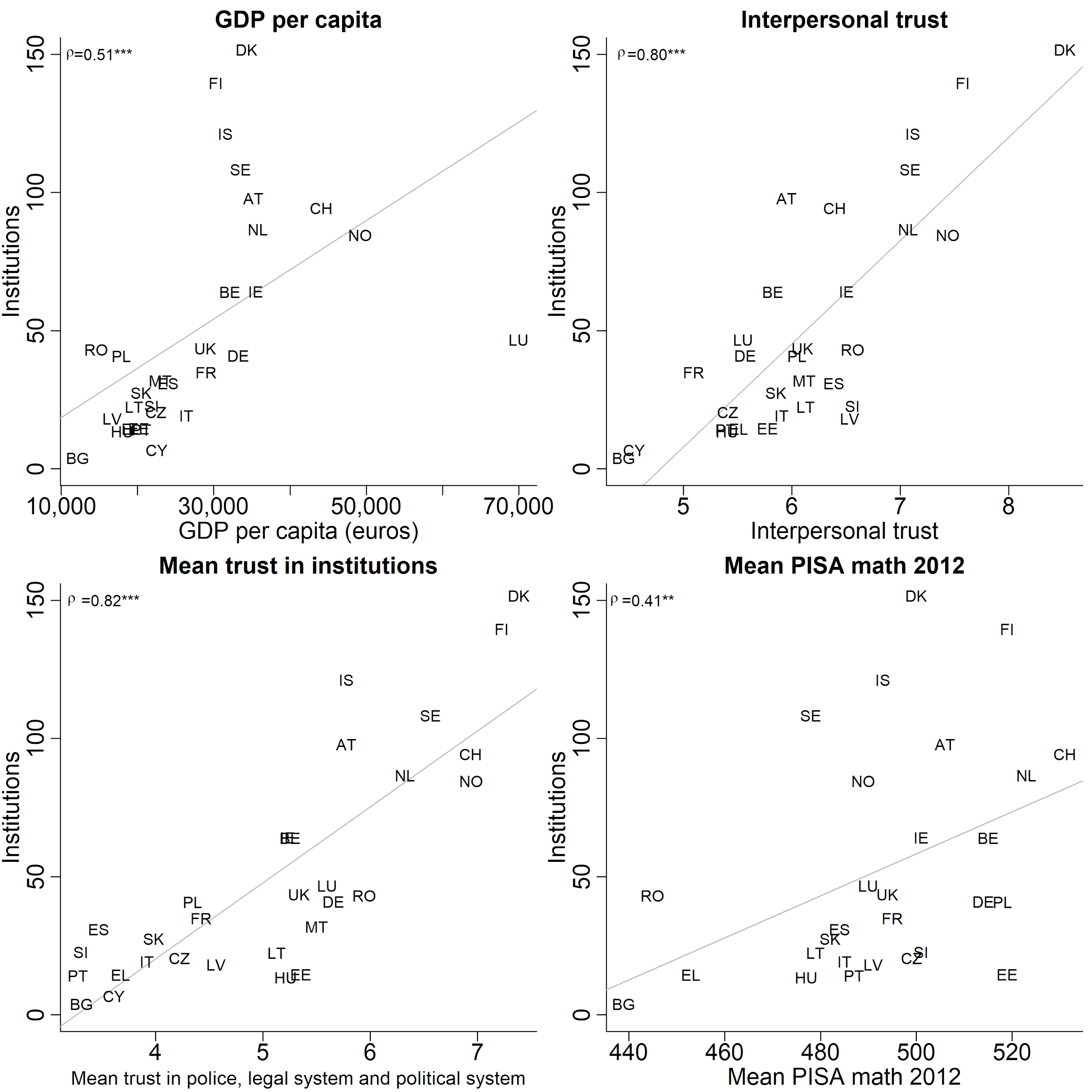
**

*Notes: Statistical significance of correlation: * p<0.1; ** p<0.05; *** p<0.01. All figures show a scatter plot and correlations for 30 countries except the top left panel, which is for 25 countries, and the bottom right panel, which is for 28 countries. Source. Income: Eurostat ppp GDP per capita. PISA math: PISA. All other sources: own calculations using EU-SILC.*

Figure A5.2 (cont.). Country-level scatter plots


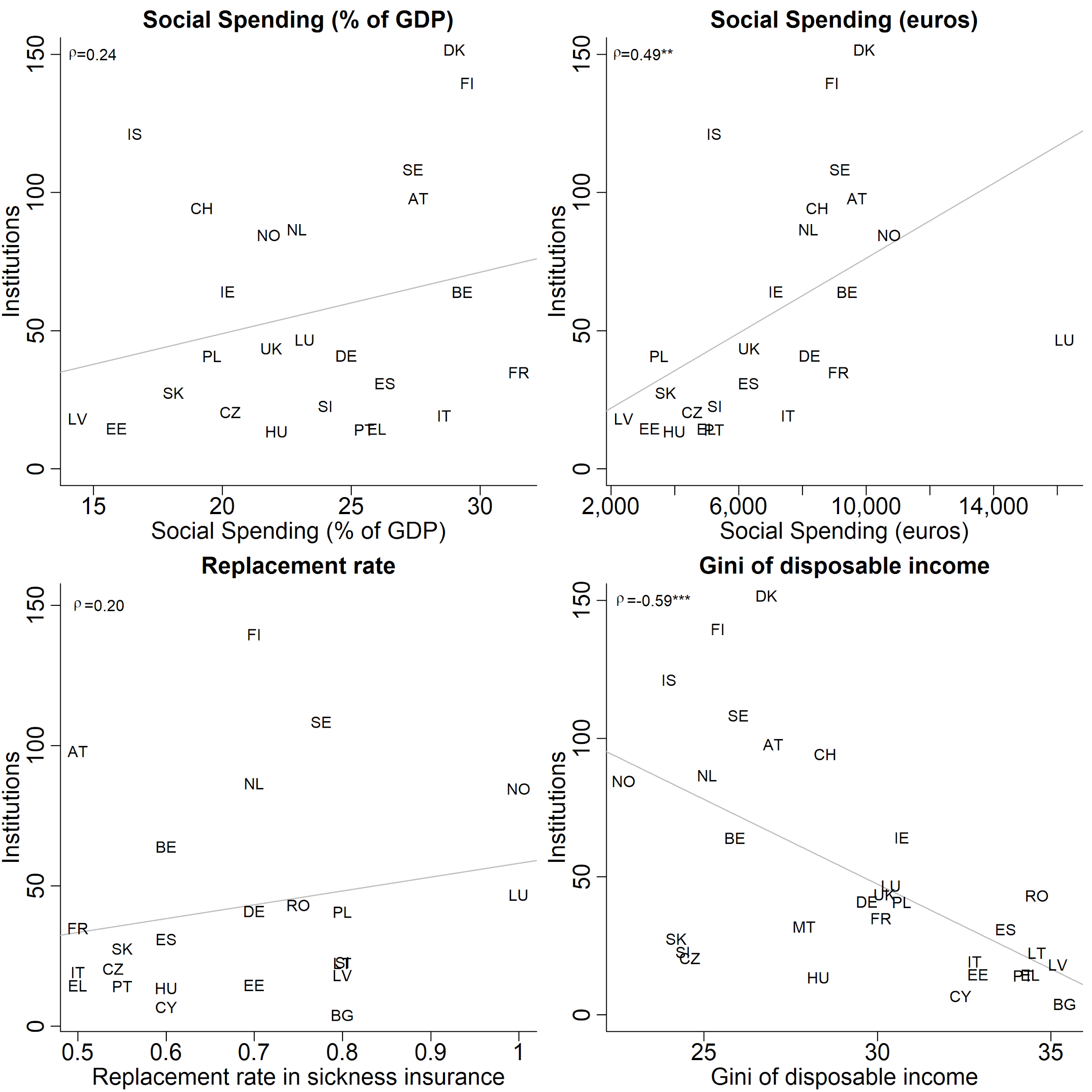


*Notes: Statistical significance of correlation: * p<0.1; ** p<0.05; *** p<0.01. All figures show a scatter plot and correlations for 25 countries in the top panels and 24 and 30 countries in the bottom left and right panels, respectively. Source. Social spending: OECD, GDP and Gini: Eurostat. Replacement rate: MISSOC (2017). All other sources: own calculations using EU-SILC.*

Table A5.2. Estimated relative contribution of the institution parameter ($\omega$) and equivalized disposable income at mean income by country

| Country | Mean life satisfaction (EU-SILC) | Estimated utility at mean GDP | Estimated utility at zero income | Contribution of income to utility | Relative contribution of institutions to utility (%) |
| --- | --- | --- | --- | --- | --- |
| Austria | 8.1 | 7.9 | 7.6 | 0.2 | 96.8 |
| Belgium | 7.8 | 7.6 | 7.2 | 0.4 | 95.0 |
| Bulgaria | 5.5 | 5.4 | 2.5 | 3.0 | 45.1 |
| Switzerland | 8.1 | 7.9 | 7.6 | 0.3 | 96.0 |
| Cyprus | 6.5 | 6.3 | 3.8 | 2.5 | 59.8 |
| Czech Republic | 7.2 | 6.8 | 5.8 | 1.0 | 85.6 |
| Germany | 7.5 | 7.4 | 6.7 | 0.6 | 91.4 |
| Denmark | 8.3 | 8.1 | 8.0 | 0.1 | 98.2 |
| Estonia | 6.8 | 6.5 | 5.3 | 1.3 | 80.6 |
| Greece | 6.6 | 6.5 | 5.2 | 1.2 | 80.8 |
| Spain | 7.4 | 7.1 | 6.4 | 0.7 | 90.4 |
| Finland | 8.3 | 8.0 | 7.9 | 0.1 | 98.2 |
| France | 7.4 | 7.2 | 6.5 | 0.7 | 90.6 |
| Hungary | 6.6 | 6.4 | 5.1 | 1.3 | 80.1 |
| Ireland | 7.8 | 7.6 | 7.2 | 0.4 | 94.6 |
| Iceland | 8.2 | 8.0 | 7.8 | 0.2 | 97.8 |
| Italy | 7.1 | 6.9 | 5.7 | 1.1 | 83.3 |
| Lithuania | 7.1 | 6.8 | 5.9 | 0.8 | 87.9 |
| Luxembourg | 7.7 | 7.8 | 6.9 | 0.9 | 88.7 |
| Latvia | 6.9 | 6.5 | 5.6 | 0.9 | 86.0 |
| Malta | 7.4 | 7.1 | 6.4 | 0.6 | 91.0 |
| Netherlands | 8.0 | 7.8 | 7.5 | 0.3 | 96.2 |
| Norway | 8.0 | 7.9 | 7.5 | 0.4 | 95.1 |
| Poland | 7.6 | 7.1 | 6.7 | 0.4 | 94.4 |
| Portugal | 6.6 | 6.5 | 5.2 | 1.3 | 79.9 |
| Romania | 7.6 | 7.1 | 6.8 | 0.3 | 95.5 |
| Sweden | 8.1 | 7.9 | 7.7 | 0.2 | 97.3 |
| Slovenia | 7.3 | 6.8 | 6.0 | 0.9 | 87.2 |
| Slovakia | 7.3 | 6.9 | 6.2 | 0.7 | 90.1 |
| United Kingdom | 7.5 | 7.3 | 6.8 | 0.5 | 92.7 |

*Notes: The values presented in columns 3–6 are based on a fit of equation (6), in which only ω and θ are allowed to vary and all other parameters are held constant at the values presented in Table A4.2.*

1. If modeled, they would add a component on the cost side. [↑](#footnote-ref-1)
2. See <http://ec.europa.eu/eurostat/web/income-and-living-conditions/overview>. The data are public and available to other researchers subject to following the EU procedures, and our method is replicable for sickness insurance and other policy domains. [↑](#footnote-ref-2)
